# Key Electronic, Linear and Nonlinear Optical Properties of Designed Disubstituted Quinoline with Carbazole Compounds

**DOI:** 10.3390/molecules26092760

**Published:** 2021-05-07

**Authors:** Bakhat Ali, Muhammad Khalid, Sumreen Asim, Muhammad Usman Khan, Zahid Iqbal, Ajaz Hussain, Riaz Hussain, Sarfraz Ahmed, Akbar Ali, Amjad Hussain, Muhammad Imran, Mohammed A. Assiri, Muhammad Fayyaz ur Rehman, Chenxi Wang, Changrui Lu

**Affiliations:** 1 Department of Chemistry, Chemical Engineering and Biotechnology, Donghua University, Shanghai 201620, China; bakhat.ali@kfueit.edu.pk; 2 Department of Chemistry, Khwaja Fareed University of Engineering & Information Technology, Rahim Yar Khan 64200, Pakistan; sumreen.asim@kfueit.edu.pk (S.A.); mrzahidiqbal77@gmail.com (Z.I.); 3 Department of Chemistry, University of Okara, Okara 56300, Pakistan; usman.chemistry@gmail.com (M.U.K.); riazhussain@uo.edu.pk (R.H.); amjadhussain@uo.edu.pk (A.H.); 4 Institute of Chemical Sciences, Bahauddin Zakariya University, Multan 60800, Pakistan; ajaz_hussain01@yahoo.com; 5 KBCMA College of Veterinary and Animal Sciences, Narowal 51600, Pakistan; sarfraz.ahmed@uvas.edu.pk; 6 Institute of Chemistry, University of Sargodha, Sargodha 40100, Pakistan; akbarchm@gmail.com (A.A.); fayyaz9@gmail.com (M.F.u.R.); 7 Department of Chemistry, Faculty of Science, King Khalid University, P.O. Box 9004, Abha 61413, Saudi Arabia; imranchemist@gmail.com (M.I.); maassiri@kku.edu.sa (M.A.A.); 8 Department of Cardiovascular Surgery, Renji Hospital, School of Medicine, Shanghai Jiaotong University, Shanghai 200240, China

**Keywords:** carbazole, quinoline, NLO response, density functional theory, acceptor units

## Abstract

Organic materials development, especially in terms of nonlinear optical (NLO) performance, has become progressively more significant owing to their rising and promising applications in potential photonic devices. Organic moieties such as carbazole and quinoline play a vital role in charge transfer applications in optoelectronics. This study reports and characterizes the donor–acceptor–donor–π–acceptor (D–A–D–π–A) configured novel designed compounds, namely, **Q3D1**–**Q3D3**, **Q4D1**–**Q1D2**, and **Q5D1**. We further analyze the structure–property relationship between the quinoline–carbazole compounds for which density functional theory (DFT) and time-dependent DFT (TDDFT) calculations were performed at the B3LYP/6-311G(d,p) level to obtain the optimized geometries, natural bonding orbital (NBO), NLO analysis, electronic properties, and absorption spectra of all mentioned compounds. The computed values of λ_max_, 364, 360, and 361 nm for **Q3**, **Q4**, and **Q5** show good agreement of their experimental values: 349, 347, and 323 nm, respectively. The designed compounds (**Q3D1**–**Q5D1**) exhibited a smaller energy gap with a maximum redshift than the reference molecules (**Q3**–**Q5**), which govern their promising NLO behavior. The NBO evaluation revealed that the extended hyperconjugation stabilizes these systems and caused a promising NLO response. The dipole polarizabilities and hyperpolarizability (β) values of **Q3D1**–**Q3D3**, **Q4D1**-**Q1D2**, and **Q5D1** exceed those of the reference **Q3**, **Q4**, and **Q5** molecules. These data suggest that the NLO active compounds, **Q3D1**–**Q3D3**, **Q4D1**–**Q1D2**, and **Q5D1**, may find their place in future hi-tech optical devices.

## 1. Introduction

Recently, the demand for more efficient optoelectronic materials has skyrocketed. Numerous theoretical and experimental research groups focus on the development of nonlinear optical (NLO) materials [1,2,3,4,5,6] owing to their overwhelming potential in photonic-based technologies [7,8], including two-photon excitation fluorescence imaging [9], multidimensional optical-based data storage devices [10], optical communication [11], and numerous other optical devices [12]. Studies have also reported different NLO active materials, including molecular dyes and organic and inorganic polymers, as well as nanomaterials [13,14,15,16,17]. Among the NLO active compounds described above, organic compounds, especially with a π-extended skeleton [18], attract more interest in NLO studies due to their efficient intra-molecular charge transfer (ICT) process. Organic compounds synthesis is relatively easily, and these compounds possess higher photothermal stability, structural flexibility, and quick response time [19,20].

Recently, the electronic properties for photonic applications have extended to many organic compounds with donor–π–acceptor (D-π-A) chemical systems where the donor (D) plays the role of hole transport and the acceptor (A) plays a role as an electron transporter [21]. Usually, non-centrosymmetric organic compounds show a second-order nonlinear polarizability response [22]. Specifically, the compounds consisting of electron donor (D) and electron acceptor (A) moieties connected by a π-conjugated bridge led to a considerable increment in the ICT process [23,24,25,26,27,28]. Consequently, the transfer of electron density from D to A units via a π-bridge imparts NLO properties to D-π-A organic compounds [29]. The literature has reported many different configurations of π-conjugated systems: D−π−A, D−A, D−π−π−A, A−π−D−π−A, D−π−A−π−D, D−D−π−A, D−A−π−π−A and D−A−π−A [30,31,32,33,34], showing an enhancement in ICT that might eventually result in a significant NLO response. One such compound is quinoline, which plays many roles in optoelectronic applications.

Quinoline and its derivatives have applications in various fields, such as organic light-emitting diode (OLED) [35] and solar cell (SC) technologies [36]. Quinoline-based compounds possess high thermal and chemical stability, electron-transporting capability, and easy structural modification. These key properties are highly desired in optoelectronic applications [37,38]. The electron-withdrawing nature of quinoline also plays a significant role in the electron transportation process. On the other hand, carbazole, with an electron-rich moiety, has many applications and is frequently used in hole-transporter materials [39,40]. Quinoline derivatives have been prepared by diverse synthetic routes, especially cross-coupling reactions using different catalysts [41,42,43]. Kiymaz et al. [44] reported that the substitution effect on a carbazole ring results in better performance of dyes with a C4 atom alkyl chain than dyes containing an alkyl chain with C2, C6, and C12 atoms. Recently, Slodek et al. [45] presented a vital synergistic experimental–computational report on how the different lengths of the alkyl chain, substituted at the 3-position (N atom) of a carbazole ring, affect the electrochemical and optical properties of the entire system. This report also confirms that different substitutions at the nitrogen (N) atom of the carbazole ring can tune the overall characteristics of compounds [45]. However, no study has reported the NLO-based properties of these synthesized molecules. Consequently, we utilized synthesized **Q3** (2,2′-(quinoline-2,4-diyl)bis(9-methyl-9H-carbazole), **Q4** (2,2′-(quinoline-2,4-diyl)bis(9-octyl-9H-carbazole), and **Q5** (2,2′-(quinoline-2,4-diyl)bis(9-decyl-9H-carbazole) compounds by Slodek et al. and designed 2,4-dicarbazolyl-substituted quinoline-based D–A–D–π–A-type novel compounds: **Q3D1**(2-((5′-(2-(4-(9-methyl-9H-carbazol-2-yl)quinolin-2-yl)-9H-carbazol-9-yl)-[2,2′-bithiophen]-5-yl)methylene)malonic acid), **Q3D2**(2-((5′-(2-(4-(9-methyl-9H-carbazol-2-yl)quinolin-2-yl)-9H-carbazol-9-yl)-[2,2′-bithiophen]-5-yl)methylene)malononitrile), **Q3D3**((E)-2-cyano-3-(5′-(2-(4-(9-methyl-9H-carbazol-2-yl)quinolin-2-yl)-9H-carbazol-9-yl)-[2,2′-bithiophen]-5-yl)acrylic acid), **Q4D1**((E)-2-cyano-3-(5′-(9-octyl-7-(4-(9-octyl-9H-carbazol-2-yl)quinolin-2-yl)-9H-carbazol-3-yl)-[2,2′-bithiophen]-5-yl)acrylic acid), **Q4D2** (2-((5′-(9-octyl-7-(4-(9-octyl-9H-carbazol-2-yl)quinolin-2-yl)-9H-carbazol-3-yl)-[2,2′-bithiophen]-5-yl)methylene)malononitrile), and **Q5D1**((E)-2-cyano-3-(5′-(9-decyl-7-(4-(9-decyl-9H-carbazol-2-yl)quinolin-2-yl)-9H-carbazol-3-yl)-[2,2′-bithiophen]-5-yl)acrylic acid). This study investigates the NLO properties of synthesized **Q3**, **Q4**, and **Q5** and their designed **Q3D1**–**Q3D3**, **Q4D1**–**Q4D2**, and **Q5D1** compounds. In a nutshell, the optimization with frequency analysis revealed that all compounds were found at a true minimum in the potential surface. Further, frontier molecular orbitals (FMOs), natural bonding orbitals (NBOs), UV/Vis analyses, and global reactivity parameters, as well as a two-level model, were performed to support the NLO results of all compounds. Furthermore, our results show that 2,4-dicarbazolyl-substituted quinoline compounds may help to develop potent NLO compounds for future hi-tech applications.

## 2. Results and Discussion

### 2.1. Modeling and Designing of Compounds

**Figure 1:**
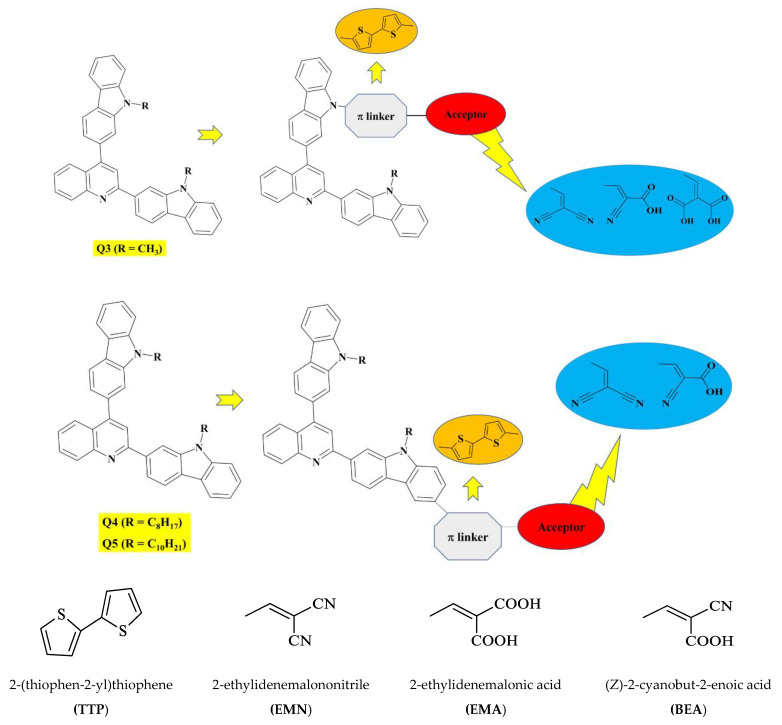
Scheme and structures of spacer and different terminal acceptors used in the designed compounds **Q3**, **Q4**, and **Q5**.

**Figure 2:**
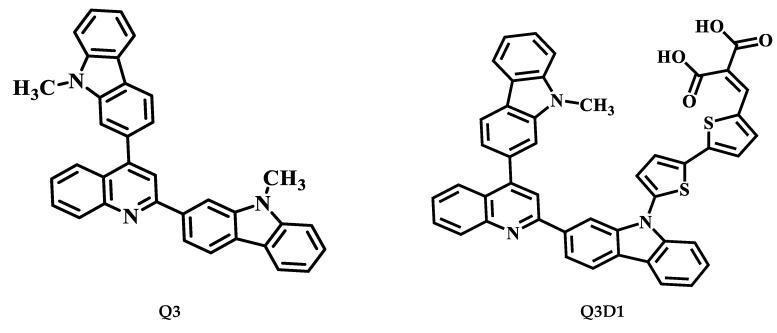

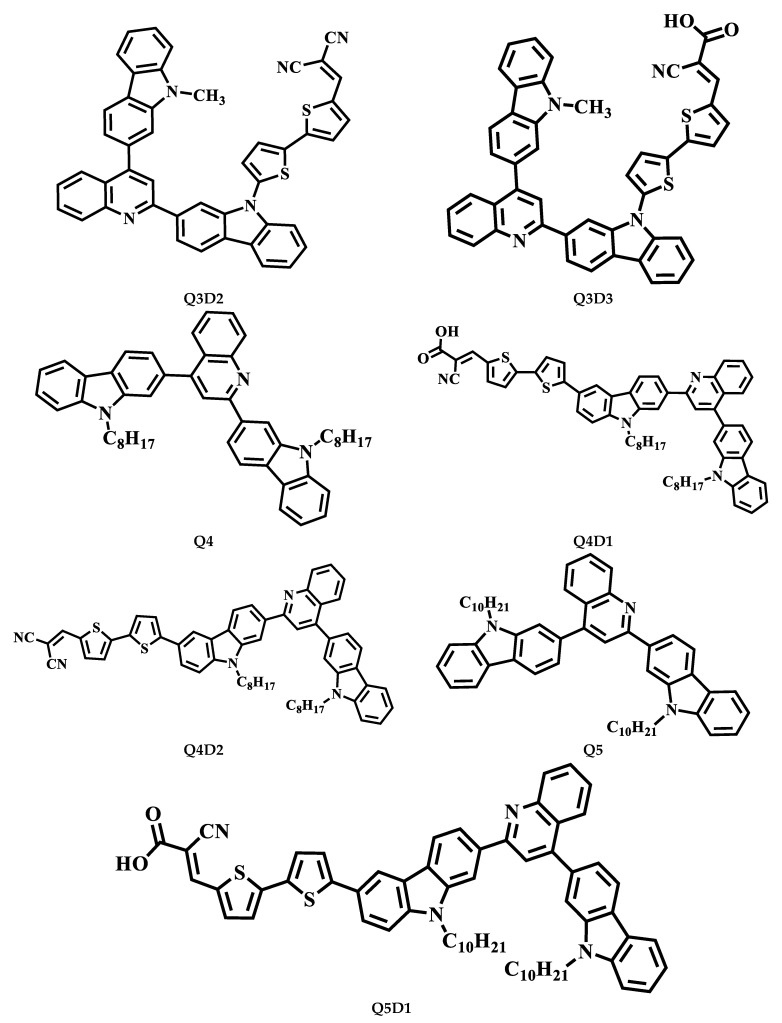
Structures of studied compounds **Q3D1**–**Q3D3, Q4D1**–**Q4D2**, and **Q5D1**.

This quantum chemical calculation work aims to analyze the NLO of novel 2,4-disubstituted quinoline–carbazole-based compounds. For designing novel organic chromophores (**Q3D1**–**Q3D3**, **Q4D1**–**Q4D2**, and **Q5D1**), recently synthesized compounds (**Q3**, **Q4**, and **Q5**) are used. These parent compounds (**Q3**, **Q4**, and **Q5**) have a donor–acceptor–donor (D-A-D) structure in which carbazole, acting as a D moiety, is present at both termini of the molecules. At the same time, quinoline is an acceptor moiety present at the center core of the molecules (Figure 1). The different electron-donating groups (alkyl groups), such as CH_3_, C_8_H_17_, and C_10_H_21_, are attached to the N atom of the carbazole (D) in **Q3**, **Q4**, and **Q5**, respectively [45]. Studies have shown that the extent of the π-conjugated system and nature of D, A moieties play a crucial role in ICT characteristics, the HOMO–LUMO energy gap and absorption spectrum, which lead to fine-tuning of the NLO response properties of materials. Both theoretical and experimental studies have shown that the significant second-order NLO response originates from the union of strong D and A groups held at the opposite ends of a proper π-conjugated system. Thus, we designed compounds from synthesized reference molecules (**Q3**, **Q4**, and **Q5**) with the addition of a donor–acceptor–donor–π–acceptor (D–A–D–π–A) model. The selected compounds, **Q3D1**, **Q3D2**, and **Q3D3**, retained the carbazole–quinoline–carbazole-based D–A–D part, while we modified the architecture of the synthesized **Q3** molecule (Figure 1) by connecting it with 2-(thiophen-2-yl) thiophene **(TTP**) as a spacer linker and different terminal units such as 2-ethylidenemalonic acid **(EMA**), 2-ethylidenemalononitrile **(EMN**), and (Z)-2-cyanobut-2-enoic acid **(BEA**) to act as acceptor units (Figure 1). Similarly, **Q4D1**–**Q4D2** structures combined the synthesized **Q4** molecule with **TTP** as a spacer and **BEA**, **EMN** as A segments (Figure 1). However, **Q5D1** was modeled by connecting the **Q5** architecture with **TTP** and **BEA** as a π-conjugated system and A moiety, respectively (Figure 1). The chemical structures of reference molecules (**Q3**, **Q4**, and **Q5**) and designed compounds (**Q3D1**–**Q3D3**, **Q4D1**–**Q4D2**, and **Q5D1**) are displayed in Figure 2. Next, we performed DFT and TDDFT computations on synthesized (**Q3**–**Q5**) and designed compounds (**Q3D1**–**Q3D3**, **Q4D1**–**Q4D2**, and **Q5D1**) to predict their NLO response properties. These calculations aimed to elucidate the definite guidelines for designing novel NLO compounds and describe how π-conjugated linkers and different A units affect the photophysical, electronic, and NLO responses. For the present probe, basic parameters such as (i) electronic properties, (ii) NBO investigation, (iii) polarizability (α), (iv) hyperpolarizability (β), (v) absorption wavelength, and (vi) light-harvesting efficiency (LHE) are calculated. We believe this work will aid future research on the synthesis of proficient NLO materials.

### 2.2. Frontier Molecular Orbital (FMO) Analysis

The frontier molecular orbital (FMO) analyses explain the chemical stability, electronic, and optical properties of understudied molecules [46]. FMOs collectively consist of a lower unoccupied molecular orbital (LUMO) and higher occupied molecular orbital (HOMO), which describe numerous interactions between conjugated systems, types of reaction, the UV–visible spectrum, and fluorescence [47]. Usually, the LUMO shows the ability to accept an electron, while the HOMO expresses the ability to donate an electron [48,49]. The E_HOMO_–E_LUMO_ explains the chemical softness, hardness, dynamic stability, and chemical reactivity of the compounds under investigation. Compounds with large E_gap_ values exist as hard compounds in nature, offering resistance to change in their electronic configuration. Conversely, compounds with a small E_gap_ value are softer, more reactive, and highly polarizable and show excellent NLO properties. The calculations for energies of the HOMO, LUMO, and energy gap of synthesized (**Q3**–**Q5**) and designed compounds **Q3D1**–**Q3D3**, **Q4D1**–**Q4D2**, **Q5D1** are shown in Table 1.

The experimental reported HOMO energy for the reference **Q3** molecule, −5.99 eV [45], corresponds to a HOMO energy value of −5.593 eV of **Q3** computed at the B3LYP/6−311G(d,p) level of theory. For **Q4**, the reported and DFT-computed HOMO energy values are −5.83 eV [45] and −5.546 eV, respectively. For **Q5**, the experimental reported value (−5.90 eV) [45] corresponds to the DFT-computed (−5.540 eV) energy value for HOMO. These results indicate that the adopted DFT method B3LYP and basis set 6−311G (d,p) combination warrants further computational calculations. From Table 1, E_gap_ values of **Q3**, **Q4**, and **Q5** are similar. Among all the synthesized molecules (**Q3**, **Q4**, and **Q5**), the **Q4** molecule with values of E_LUMO_ of −1.768 eV and E_HOMO_ of −5.546 eV has the largest E_gap_ value, while **Q5** has the lowest E_gap_ value of 3.775 eV. Table 1 shows that all synthesized reported compounds (**Q3**, **Q4**, and **Q5**) have a larger E_gap_ value than their designed compounds. Next, we examine the gap reduction in the designed species. In **Q3** and its derivatives, **Q3** has the highest value of E_gap_ (3.777 eV) that dwindled to 3.047 eV in **Q3D1**. This 0.73 eV reduction in the E_gap_ value of **Q3D1** might result from the combined effect of extended conjugation of **TTP** spacers and the electron-withdrawing effect of **EMA** containing two COOH groups present in **Q3D1** (absent in **Q3**). The E_gap_ value was further reduced to 2.157 eV in **Q3D2**. The **TTP** spacer linker and two CN groups present in the **EMN** terminal acceptor collectively reduce the large energy gap in **Q3D2** compared to **Q3**. A similar effect was observed in **Q3D3**, where CN, COOH groups present in the **BEA** acceptor and **TTP** spacer reduced the E_gap_ value to 2.313 eV (1.464 eV less compared to **Q3**). The E_gap_ order of **Q3** and its derivatives is **Q3D2** < **Q3D3** < **Q3D1** < **Q3**, which suggests the effectiveness of terminal acceptors in the order of **EMN** > **BEA** > **EMA**. A similar effect holds true for **Q4** and its derivatives. **Q4**’s value of 3.778 eV, the greatest energy gap value, decreased to 2.594 eV in **Q4D1** and then further decreased to 2.492 eV in **Q4D2**, reductions of 1.184 eV and 1.286 eV, respectively. The sharp narrowing of the energy gap in **Q4D1** and **Q4D2** results from the effect of **TTP** spacers and **BEA**, **EMN** terminal acceptor units present in **Q4D1** and **Q4D2** architectures which are absent in **Q4**. The efficiency in bridging the energy gap increases for **EMN** in **Q4D2** over **BEA** in **Q4D1**. Therefore, the increasing E_gap_ order of **Q4** and its derivatives is **Q4D2** < **Q4D1** < **Q4**. Among **Q5** and its derivative **Q5D1**, **Q5**’s largest value of E_gap_ of 3.775 eV narrows down to 2.609 eV in **Q5D1** due to the inclusion of **TTP** and **BEA** as the π-conjugated system and A moiety. The final increasing order for the energy gap ranks as follows: **Q3D2** < **Q3D3** < **Q4D2** < **Q4D1** < **Q5D1** < **Q3D1** < **Q5** < **Q3** < **Q4**. This reduction in the energy gap of the designed derivatives compared to synthesized (reported) compounds shows the effect of structural tailoring by introducing planar, electron-rich π-linkers and different suitable terminal acceptor units. These modifications can tailor the molecules to exhibit desired NLO characteristics.

**Figure 3:**
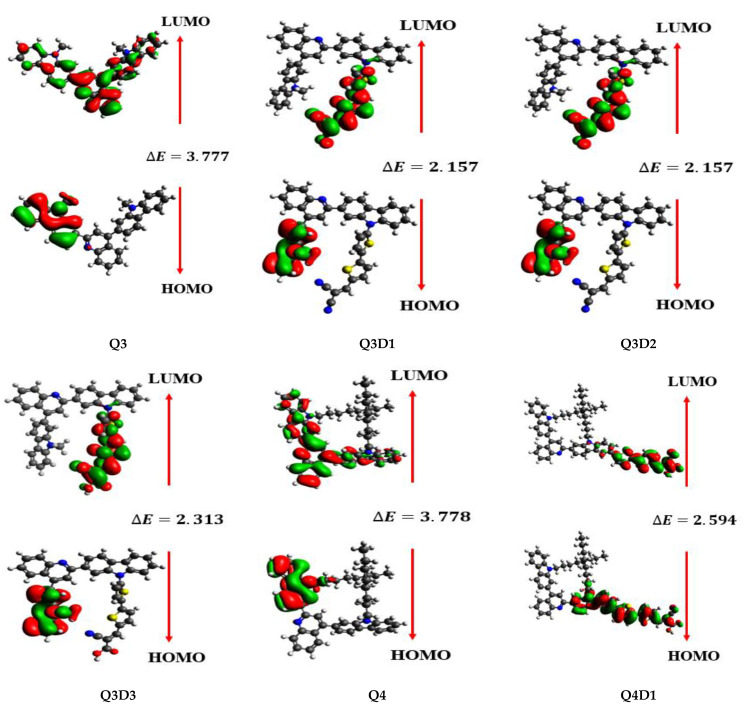

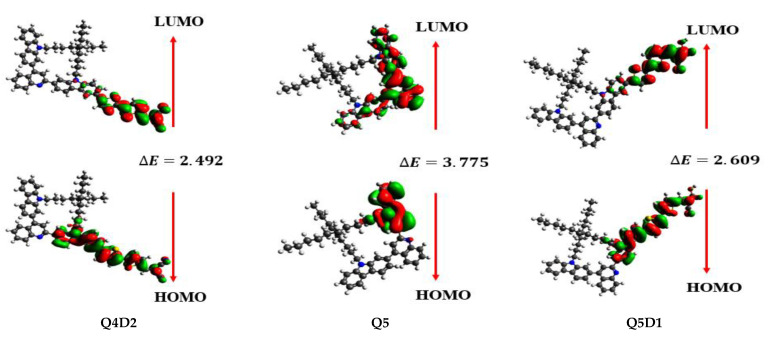
HOMOs and LUMOs of the studied compounds.

Figure 3 shows the pictorial display of the HOMO and LUMO of synthesized (**Q3**–**Q5**) and designed compounds (**Q3D1**–**Q3D3**, **Q4D1**–**Q4D2**, and **Q5D1**). Most of the HOMO electron charge separation spread over the donor carbazole part of the compounds compared to the bridge part. Meanwhile, the LUMO largely spread over the terminal acceptors’ units and partially on the π-spacer of the studied compounds. This analysis suggests that the charge shifted from a D moiety to A unit via π-linkers and demonstrates that these materials can have desired NLO properties.

### 2.3. Global Reactivity Parameters (GRPs)

The strength of FMOs (E_gap_ = E_LUMO_–E_HOMO_) includes the global reactivity information, such as global hardness (η), global softness (S), global electrophilicity index (ω) electron affinity (EA), ionization potential (IP), electronegativity (X), and the chemical potential (μ). Equations (7)–(13) are utilized to calculate the global reactivity parameters (GRPs), and results are given in Table 2.

The GRPs illustrated the reactivity of **Q3**–**Q5** (synthesized) and **Q3D1**–**Q5D1** (designed) compounds. Ionization potential expresses the electron-donating ability and equals the energy compulsory to extract one electron from the HOMO. Electronegativity helps to explain the propensity of electron cloud interactions. Another chemical parameter, chemical potential, relates to the reactivity and stability of compounds. A direct relation between energy gap, hardness, chemical potential, and stability collectively contributes towards an inverse relationship towards chemical reactivity. Hence, molecules with a larger energy gap will show more kinetic stability, lower reactivity, and more resistance to any electronic configuration change. The value of E_gap_ is highest in **Q4** among the synthesized compounds (**Q3**, **Q4**, and **Q5**). The hardness value calculated for **Q4** is higher (1.889) with a chemical reactivity of −3.657. Meanwhile, **Q5** has the lowest value of calculated hardness of 1.887, and **Q3** has a hardness value of 1.888, with a chemical reactivity of −3.652 and −3.701 for **Q5** and **Q3**, respectively. These results show that **Q4** is less reactive and more chemically stable than **Q3** and **Q5**.

The **Q3** derivatives show reduced hardness values compared to native **Q3**. The calculated value of hardness of 1.523 in **Q3D1** decreased to 1.156 in **Q3D3** and 1.078 in **Q3D2**. Similarly, Table 2 shows the diminishing chemical potential values. Among all the compounds studied, **Q3D2** is the least chemically stable with the smallest calculated value of hardness and a minimum chemical potential value of −4.519. Similarly, the examined hardness and the potential chemical value also decreased in **Q4** derivatives (Table 2). The hardness value for **Q4D1** (1.297) further declined to 1.246 in **Q4D2**. Amongst all **Q4** derivatives, **Q4D2** showed the smallest chemical potential value of −4.372 and, hence, the least stability. The designed compounds of **Q5** also showed diminishing hardness and chemical potential values. The decreasing order of hardness of all investigated compounds ranks as follows: **Q4** > **Q3** > **Q5** > **Q3D1** > **Q5D1** > **Q4D1** > **Q4D2** > **Q3D3** > **Q3D2**, similar to the HOMO–LUMO energy gap order.

Next, we evaluated softness which directly relates to the chemical reactivity of the molecules under investigation. The value of global softness is 0.264 in the reference compounds **Q3**, **Q4**, and **Q5**. Our designed compounds display extended softness values. In the designed compounds of **Q3**, **Q3D1** expressed a value of 0.328 of softness, while **Q3D3** shows 0.432 and **Q3D2** further increases to 0.463. Similarly, among **Q4** derivative molecules, **Q4D1** showed a 0.385 value of global softness, which increases to 0.401 in **Q4D2**. This result shows that **Q4D2** has the most reactivity. The value of softness also increased in the designed compound **Q5D1** compared to its parent molecule **Q5**. From all investigated molecules, **Q3D2** has the highest value of softness, thus the highest chemical reactivity. The decreasing order of softness among all the investigated molecules ranks as exactly the inverse to the increasing E_gap_ order: **Q3D2** > **Q3D3** > **Q4D2** > **Q4D1** > **Q5D1**> **Q3D1** > **Q4** > **Q3** > **Q5**. All these global reactivity parameter investigations illustrate that the investigated compounds might hold promising NLO characteristics.

### 2.4. Natural Bond Orbital (NBO) Analysis

The NBO study provides the Lewis structures, atomic charge, hybridization, and diverse bonds (dative, ionic, covalent, σ and π) [28,50]. The stabilization energy E^(2)^ with a second-order perturbation method is calculated using Equation (10).

(1) $$E^{\left(2\right)}{=q}_{i}\frac{{{(F}_{i,j})}^{2}}{\varepsilon_{j}{-\varepsilon }_{i}}$$

where q_i_ means occupancy of contributor orbital, έ_j_ and έ_i_ are the off-diagonal NBO Fock matrix elements, F(i,j) is the diagonal, and E^(2)^ is stabilization energy.

In this NBO study, electron acceptor–donor interactions are shown by the E^(2)^ value. Table S1 (Supplementary Information) shows hyperconjugative interactions of the acceptor moieties and electron-contributing moieties of the reference and designed compounds. We show that four major transitions, σ→σ*, π→π*, LP→σ*, and LP→π*, are observed for all the chemical compounds. The (π→π*) transitions were extensively observed with a large E^(2)^, whereas LP→σ* and LP→π* transitions were found with a small E^(2)^ in comparison to π→π*. In addition, the lowest E^(2)^ values originate from σ→σ* transitions.

The electronic π→π* transitions of peak values are π(C8–C12)→ π*(C11-N23), π(C70–C72)→π*(C74–C76), π(C8–C12)→π*(C11–N23), and π(C70–C72)→π*(C74–C76), with stabilization energy of 24.1, 24.53, 24.90, and 25.75 kcal/mol in **Q3**, **Q3D1**, **Q3D2**, and **Q3D3**, respectively. Some transitions with less stabilization energy were also observed, including π(C35–C36)→π*(C8–C12), π(C8–C12)→π*(C35–C36), π(C8–C12)→π*(C35–C36), and π(C8–N12)→π*(C3–C4), with 5.15, 5.58, 6.04, and 13.68 kcal/mol in **Q3**, **Q3D1**, **Q3D2**, and **Q3D3**, respectively.

In these compounds, transitions in accordance with the σ→σ* transitions were: σ(C26–C27)→σ*(N24–C25), σ(C74–C75)→σ*(S69–C72), σ(C76–C77)→σ*(C77–N80), and σ(C76-C77)→σ*(C77-N80). These interactions contribute the most among all σ→σ* transitions, with stabilization energy of 6.16, 9.45, 8.68, and 8.62 kcal/mol in **Q3** and its derivative compounds (**Q3D1**–**Q3D3**), respectively. Some transitions with minimal energy are: σ(C27–H32)→σ*(C27–C28), σ(C12–H13)→σ*(C3–C8), σ(C28–C29)→σ*(C21–C30) and σ(C60–S62)→σ*(C60–C61), with 0.54, 5.03, 5.01, and 0.55 kcal/mol in **Q3**, **Q3D1**, **Q3D2**, and **Q3D3,** respectively.

Moreover, we observed LP→π* transitions: LP(N54)→π*(C38–C42), LP2(O80)→π*(C77–O79), LP1(N54)→π*(C38–C42), and LP2(O80)→π*(C77–O79), with 36.87, 45.85, 37.92, and 45.47 kcal/mol stabilization energy in **Q3**, **Q3D1**, **Q3D2**, and **Q3D3**, respectively. These were the highest values of excitations from LP→π* transitions. Furthermore, we saw a small amount of stabilization energy in LP(N54)→$\partial^{*}$(C56–H59), LP1(N23)→$\partial^{*}$(C11–O12), LP1(N23)→$\partial^{*}$(C11–O12), and LP1(S62)→$\partial^{*}$(C60–C61), with 7.29, 10.53, 10.67, and 21.62 kcal/mol in **Q3** and its derivative compounds (**Q3D1**-**Q3D3**), respectively.

Similarly, in the case of **Q4**, **Q5** and their designed molecules (**Q4D1**, **Q4D2**, and **Q5D1**), we calculated π→π* electronic interactions: π(C8–C12)→π*(C11–N23), π(C8–C12)→π*(C11–C23), π(C14–C16)→π*(C19–C21), π(C8–C12)→π*(C11–C23), and π(C4–C5)→π*(C1–C6), with the highest stabilization energy of 24.23, 24.17, 21.69, 24.71, and 24.64 kcal/mol in **Q4**, **Q5**, **Q4D1**, **Q4D2**, and **Q5D1**, respectively. Meanwhile, transitions with less stabilization energy were also detected: π(C11–N23)→π*(C14–C16), π(C11–N23)→π*(C14–C16), π(C8–C12)→π*(C3–C4), π(C8–C12)→π*(C3–C4), and π(C8–C12)→ π*(C3–C4), with 8.31, 8.58, 13.57, 13.57, and 13.57 kcal/mol in **Q4**, **Q5** and their designed molecules (**Q4D1**, **Q4D2**, and **Q5D1**). Among the σ→σ* transitions, these molecules also showed transitions of σ(C47–C50)→σ*(C45–N54), σ(C47–C50)→σ*(C45–N54), σ(C106–C108)→σ*(C110–N112), σ(C26–C27)→σ*(N24–C25), and σ(C46–C49)→σ*(C44–N53), with the highest stabilization energy of 6.15, 6.13, 5.49, 5.80, and 6.13 kcal/mol in **Q4**, **Q5** and their designed compounds **Q4D1**, **Q4D2**, and **Q5D1**, respectively. The lowest stabilization energy transitions were σ(C68–C71)→σ*(C71–H73), σ(C68–C71)→σ*(C71–H73), σ(C73–H76)→σ*(C73–C74), σ(C73–H76)→σ*(C73–C74), and σ(C73–H76)→σ*(C73–C74), with 0.50 kcal/mol in **Q4**, **Q5** and their designed molecules (**Q4D1**, **Q4D2**, and **Q5D1**), respectively.

Last, but not least, the highest stabilization energy by the transitions by lone pairs LP1 (N54)→π*(C38–C42), LP1(N54)→π*(C38–C42), LP2(O123)→π*(C122–O123), LP1(N24)→π* (C25–C30), and LP2(O138)→π*(C136–C137) gave 37.08, 37.07, 41.89, 38.24, and 37.97 kcal/mol in **Q4**, **Q5**, **Q4D1**, **Q4D2**, and **Q5D1**, respectively. All these transitions had the highest stabilization energy among all transitions. The lowest value transitions were LP1 (N54)→$\partial^{*}$(C56–C62), LP1 (N54)→$\partial^{*}$(C56–C62), LP1(N23)→π*(C3–C4), LP1(N23)→π*(C3–C4), and LP1(N23)→π*(C3–C4), with 6.36, 6.33, 10.37, 10.36, and 10.37 kcal/mol in **Q4**, **Q5** and their designed molecules (**Q4D1**, **Q4D2**, and **Q5D1**), respectively.

In conclusion, our NBO calculations show that intra-molecular interactions and extended hyperconjugation in the studied compounds provide more stability and a vital explanation of charge transfer properties. Hence, they might be useful for potential NLO features.

### 2.5. Nonlinear Optical (NLO) Properties

NLO compounds widely occur in signal processing, optical switches, communication technology, and optical memory devices. The polarizability or α (linear response) and hyperpolarizabilities or β (nonlinear response) relate to the optical response generated by the electrical characteristics of the compounds under investigation. We investigated both linear and nonlinear responses of quinoline–carbazole synthesized (**Q3**–**Q5**) and designed compounds (**Q3D1**–**Q3D3**, **Q4D1**–**Q4D2**, **Q5D1**) using the B3LYP/6-311G (d,p) functional. Table 3 and Table 4 summarize the results for <α> and β values.

Table 3 represents transitions predominantly contributing to average polarizability <α> values in all studied molecules observed along the x-axis (α_xx_). The <α> value of **Q3**, computed at 477.794 (a.u), was found to be the smallest, while the largest value of 648.851 (a.u) belongs to **Q5** among all synthesized compounds (**Q3**–**Q5**). With the inclusion of different acceptor units and linker/spacer units, the designed species display increased polarizability <α> values. Specifically, structural modeling of **Q3** by incorporating **TTP** spacers and electron-withdrawing units **EMA**, **EMN**, **BEA** in **Q3D1**-**Q3D3** augmented the average polarizability in designed compounds compared to **Q3**. The <α> value increased from 477.794 (a.u) in **Q3** to 689.629 (a.u) in **Q3D1**, 696.513 (a.u) in **Q3D3**, and 703.223 (a.u) in **Q3D2** as their bandgap decreased. The order of <α> values in **Q3** and its derivatives rank as follows: **Q3D2** > **Q3D3** > **Q3D1** > **Q3**. We observed similar enhancement in **Q4** and its designed compounds. The lowest value of average polarizability, 635.500 (a.u) in **Q4**, increased to 1010.249 (a.u) in **Q4D1** and 1010.856 (a.u) in **Q4D2**. The increase in the <α> value in **Q4D1** and **Q4D2** results from the effect of **TTP** spacers and **BEA**, **EMN** terminal acceptor units present in **Q4D1**-**Q4D2** architectures and their absence in **Q4**. The increasing order for <α> values of **Q4** and its designed molecules is **Q4** < **Q4D1** < **Q4D2**. Similarly, the derivative of **Q5** exhibits the largest value of <α> of 684.851 (a.u). Among all these investigated compounds, **Q5D1** exhibits the largest value of average polarizability of 1048.496 (a.u). Overall, the decreasing order of average polarizability value of studied molecules ranks as follows: **Q5D1** > **Q4D2** > **Q4D1** > **Q3D2** > **Q3D3** > **Q3D1** > **Q5** > **Q4** > **Q3**.

Studies show that large β values correspond to narrow bandgap and large linear polarizability values [51,52]. The polarizability values are computed by employing y- or x-axis direction electronic transitions using Equation (2) (along x-direction).

(2) $$\alpha \propto \frac{{\left(M_{X}^{\mathrm{gm}}\right)}^{2}}{E_{\mathrm{gm}}}$$

In this equation, $M_{X}^{\mathrm{gm}}$ indicates the ground and m^th^ excited state transition moment. E_gm_ denotes transition energy. Equation (11) explains that α is directly proportional to the square of the transition moment and inversely proportional to transition energy. The square of the transition moment explains the power of interaction because of the distribution of charge density contained in the system. In general, a molecule with a large value of $M_{X}^{\mathrm{gm}}$ and a smaller value of E_gm_ will have a high hyperpolarizability value. Therefore, dipole polarizability quantitatively estimates the NLO response properties of compounds. Additionally, second-order polarizability or first hyperpolarizability (β) helps to compute the NLO response of materials. Table 4 shows the hyperpolarizabilities of synthesized (**Q3**–**Q5**) and designed compounds (**Q3D1**–**Q3D3**, **Q4D1**–**Q4D2**, and **Q5D1**) with β_tot_ values, along with their major contributing tensors.

Table 5 shows that the NLO response in these compounds mainly results from the x-axis tensor β_xxx_ containing larger values and contributing predominantly toward β_tot_ among all contributing tensors. All the synthesized compounds showed smaller values of β_tot_ than their designed derivatives. Among the synthesized compounds **(Q3**–**Q5**), **Q3** exhibits the highest β_tot_ value of 3277.62 (a.u), while **Q4** exhibits the lowest β_tot_ value of 3000.35 (a.u). The decreasing order of β_tot_ values for reference compounds is **Q3** > **Q5** > **Q4**. The designed molecule **Q3D1** shows an increase in hyperpolarizability value from 3277.62 (a.u) in **Q3** to 7811.75 (a.u). **Q3D3** and **Q3D2** further increased the value to 13,095.90 (a.u) and 16,375.60 (a.u). This increase in β_tot_ values compared to reference **Q3** results from the insertion of **TTP** spacers and the electron-withdrawing effect of **EMA** containing two COOH groups present in **Q3D1**, two CN groups present in the **EMN** terminal acceptor of **Q3D2**, and CN, COOH groups present in the **BEA** acceptor of **Q3D3**. The result of the terminal acceptor enlarging the nonlinear response (in the order **EMN** > **BEA** > **EMA**) corresponds to the increase in the linear response and reduction in the energy gap. We observed similar phenomena for **Q4** and **Q5** derivatives. **Q4** has the smallest value of 3000.35 (a.u). **Q4D1** increases the value to 52,662.1 (a.u) due to the **TTP** spacer and **BEA** terminal acceptor unit effect. The **TTP** spacer and **EMN** terminal acceptor present in **Q4D2** further enlarges the β_tot_ value to 59,316.4 (a.u) in **Q4D2**. The **Q5D1** derivatives containing **TTP** and **BEA** as a π-conjugated system and A moiety exhibited the higher value of hyperpolarizability of 50,156.00 (a.u) than their parent molecule **Q5** (3103.51(a.u)). Overall, **Q4D2** came first with the highest β_t__ot_ value of 69,791.4 among all investigated compounds. The compound rank in decreasing order of β_tot_ values is as follows: **Q4D2** > **Q4D1** > **Q5D1** > **Q3D2** > **Q3D3** > **Q3D1** > **Q3** > **Q5** > **Q4**.

Our β_tot_ findings for the investigated molecules are further strengthened by comparing them with an organic reference urea molecule [53]. All synthesized (**Q3**–**Q5**) and designed compounds (**Q3D1**–**Q3D3**, **Q4D1**–**Q4D2**, **Q5D1**) have greater β_tot_ values than a urea molecule, indicating the potential NLO applicability of these compounds in future NLO applications.

### 2.6. UV–Vis Spectra Analysis

**Figure 4:**
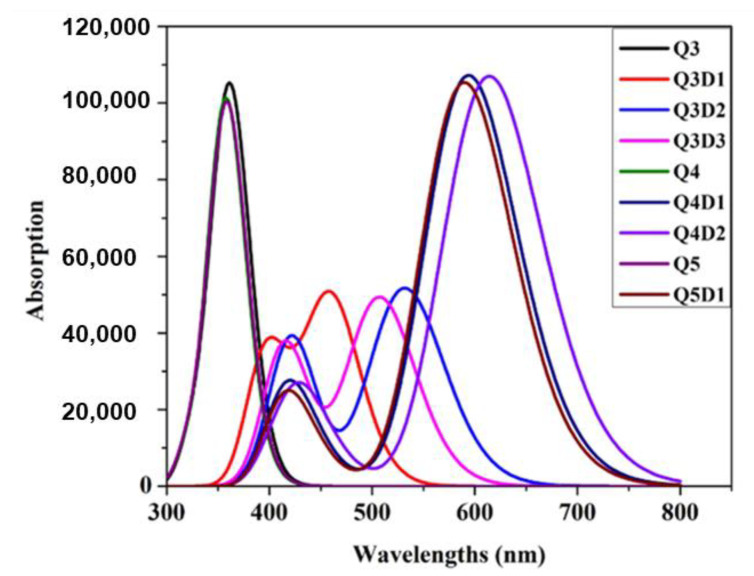
Simulated absorption spectra of studied compounds.

UV–visible spectroscopy explains the charge transfer of compounds under investigation. The absorption spectra of synthesized **Q3**-**Q5** and designed compounds **Q3D1**–**Q3D3**, **Q4D1**–**Q4D2**, **Q5D1** are computed using TDDFT at the B3LYP/6-311G(d,p) level of theory. During TDDFT computations, the six lowest singlet−singlet transitions were studied. Table 5 summarizes the results obtained from UV–visible spectral analysis, while Figure 4 shows the absorption spectra of the studied compounds.

The DFT-computed λ_max_ value of **Q3** (364.970 nm) corresponds to the experimental λ_max_ value reported at 349 nm [45]. Similarly, DFT-computed λ_max_ values of **Q4** and **Q5** (360.901 and 361.670 nm, respectively) also correspond to reported λ_max_ values at 347 and 323 nm [45]. These matches validate our selection of the B3LY/6-311G(d,p) functional for the calculations. Among all synthesized (**Q3**–**Q5**) compounds, **Q4** shows the lowest λ_max_ value of 360.90 nm, which is the lowest calculated λ_max,_ while **Q3** exhibits the highest value of maximum absorption of 364.97 nm. This shows that **Q3** leans towards the bathochromic shift. All the designed molecules exhibited a larger value of maximum absorption than their parent compounds. The absorption maximum observed in the **Q3** family reduces in transition energy, owing to the combined effect of **TTP** spacers and electron-withdrawing units **EMA**, **EMN**, **BEA** present in **Q3D1**–**Q3D3**. Consequently, λ_max_ values of **Q3D1**–**Q3D3** are increased by 95 nm, 167 nm, and 143 nm, respectively, compared to **Q3**. **Q3D2** exhibits the largest λ_max_ value of 531.57 nm with the smallest transition energy value of 2.332eV among the **Q3** family; hence, it is the most redshifted among **Q3** and its designed derivatives. The decreasing order of λ_max_ found in **Q3** and its compounds is **Q3D2** > **Q3D3** > **Q3D1** > **Q3**. We observed a similar redshift in **Q4** designed derivatives due to similar structural modifications. **Q4D1** and **Q4D2** show an increase in absorption wavelengths at 593.82 nm and 613.87 nm compared to **Q4**, caused by the combined effect of **TTP** spacers and **BEA**, **EMN** terminal acceptor units present in **Q4D1** and **Q4D2** architectures, while they are absent in **Q4**. Similar to the **Q4** family, **Q5D1** has a higher value of maximum absorption at 589.61 nm with a lower value of transition energy, 2.102eV, than **Q5**. This result shows that **Q5D1** and **Q5** are bathochromic and hypochromic, respectively. Among all the studied compounds, **Q4D2** ranks highest in maximum absorption wavelength. The decreasing order for λ_max_ values of studied compounds is: **Q4D2** > **Q4D1** > **Q5D1** > **Q3D2** > **Q3D3** > **Q3D1** > **Q3** > **Q5** > **Q4**. Table 5 indicates that major transitions among synthesized and reported molecules **Q3**, **Q4**, and **Q5** arose from HOMO-2→LUMO (≥92%). In the designed derivatives of **Q4** and **Q5**, HOMO→LUMO (99%) transitions were observed and **Q3** derivatives containing HOMO-1→LUMO (≥95%) transitions were observed. These characteristics render these compounds suitable for fine optoelectronic roles.

Excitation energy further enhances the electro-optic properties of NLO materials. The combined effect of **TTP** spacers and electron-withdrawing units **EMA**, **EMN**, **BEA** lowers the excitation energy value from 3.397 eV (**Q3**) to 2.699 eV, 2.332 eV, and 2.445 eV in **Q3D1**-**Q3D3**. In **Q4D1** and **Q4D2**, excitation energy values decrease to 2.087eV and 2.019 eV compared to the **Q4** value of 3.435eV due to the **BEA**, **EMN** terminal acceptors and **TTP** spacers present in **Q4D1** and **Q4D2**. A similar effect was observed in **Q5D1**, where the excitation energy value, 2.102 eV, is lower than that of the parent **Q5** excitation energy value of 3.428 eV. The lower excitation energy results in higher charge transfers and vice versa. Our all designed molecules exhibit low excitation energy compared to their parent compounds, ideal for NLO response properties.

Lastly, we quantified the optical efficiency of studied compounds by light-harvesting efficiency (LHE). We measured the photocurrent response of compounds by LHE using the f_os_ value and Equation (3) [54].

LHE = 1–10^−f^ (3)

Table 5 reveals that **Q3D1** has the lowest LHE value, while compounds like **Q4D1**, **Q4D2**, and **Q5D1** have large LHE values.

We opted to explain the donor–acceptor unit effect on enhanced NLO characteristics by structure–property relationships. Chemla and Oudar [55] expressed the relationship between hyperpolarizability and electronic charge transfer transition using a two-level model based on complex sum-over-states (SOS):

(4) $$\beta_{\mathrm{CT}}=\frac{{\Delta \mu }_{\mathrm{gm}}f_{\mathrm{gm}}}{E_{\mathrm{gm}}^{3}}$$

Here,${\;E}_{\mathrm{gm}}^{3}$, ∆μ_gm_, β_CT_, and f_gm_ are the most crucial m^th^ excited state excitation energy difference, dipole moment difference, first hyperpolarizability, and oscillator strength. These parameters are governed by the choice of suitable acceptor units, bridges, and inter-linked donors. A larger β value can result from the most promising blend of these parameters. The product of ∆μ_gm_ and f_gm_ has a direct relation, as seen from Equation (4), which shows an inverse relationship between the cube of transition energy and β_CT_. Thus, large ∆μ_gm_, f_gm_ magnitude, and low $E_{\mathrm{gm}}^{3}$ serve as vital gauges for the optimum design of proficient NLO compounds. From our spectral analysis, the values of ∆μ_gm_, f_gm,_ and $E_{\mathrm{gm}}^{3}$ are calculated, and results are given in Table 5.

**Figure 5:**
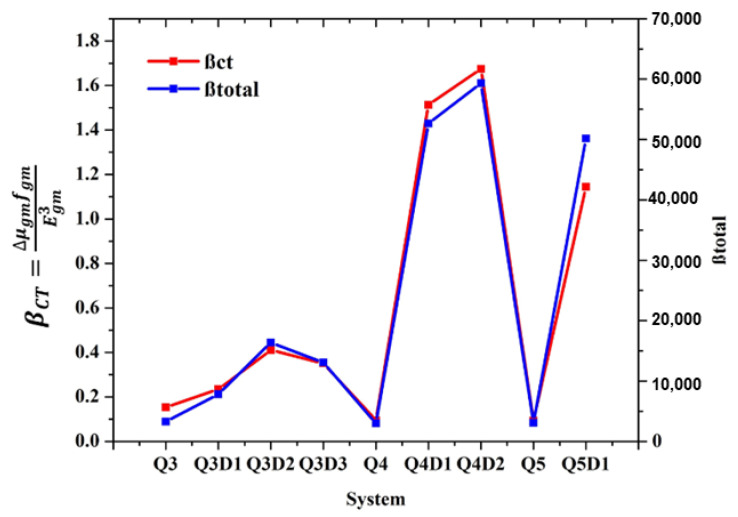
Simulated absorption spectra of quinoline–carbazole based compound (**Q3**–**Q5D1**).

A graphical representation of a comparison between the computed β_to__t_ findings and β_CT_ results (obtained from a two-level model) for synthesized (**Q3**–**Q5**) and designed compounds (**Q3D1**–**Q3D3**, **Q4D1**–**Q4D2**, **Q5D1**) is shown in Figure 5. The x-axis describes the studied systems, and the vertical y-axis indicates the magnitudes of β_tot_ and β_C__T_ results. It is evident from Figure 5 that both β_t__ot_ and β_CT_ findings are in direct relation with each other. The preceding discussion concluded that β_tot_ results are also confirmed by a two-level model, which implies that studied molecules can be recommended for future construction of high-performance NLO materials.

## 3. Computational Procedure

The Gaussian 09 program package [56] was applied to conduct all quantum chemical calculations in this study. All input files were generated using the GaussView 5.0 [57] program. All synthesized (**Q3**, **Q4**, and **Q5**) and designed chemical systems (**Q3D1**–**Q3D3**, **Q4D1**–**Q4D2**, and **Q5D1**) were optimized by employing density functional theory (DFT) computations. The B3LYP functional was utilized with a 6-311G(d, p) basis set for optimizing geometries, frequency analysis, NBO analysis, and computing the NLO parameters of entitled compounds. Time-dependent DFT (TDDFT) calculations with the B3LYP/6-311G(d,p) level was carried out for frontier molecular orbital (FMO) analysis and UV/Vis spectra estimation. Symmetry constraints were not adopted in all DFT- and TDDFT-based calculations. Six linear polarizability tensors, α_xx_, α_yy_, α_zz_, α_xy_, α_xz_, and α_yz_, and ten hyperpolarizability tensors, β_xxx_, β_xyy_, β_xzz_, β_yyy_, β_xxy_, β_yzz_, β_zzz_, β_xxz_, β_yyz_, and β_xyz_, along x-, y-, and z-directions, were collected from the Gaussian 09 output. The amplitudes of average polarizability <α> and first hyperpolarizability (β_tot_) are calculated by Equations (5) and (6) [58].

(5) $${<\alpha >=1/3(\alpha }_{\mathrm{xx}}{+\alpha }_{\mathrm{yy}}{+\alpha }_{\mathrm{zz}})$$

(6) $$\beta_{\mathrm{tot}}{=[(\beta }_{\mathrm{xxx}}{+\beta }_{\mathrm{xyy}}{+\beta }_{\mathrm{xzz}}{)}^{2}{+(\beta }_{\mathrm{yyy}}{+\beta }_{\mathrm{xxy}}{+\beta }_{\mathrm{yzz}}{)}^{2}{+(\beta }_{\mathrm{zzz}}{+\beta }_{\mathrm{xxz}}{+\beta }_{\mathrm{yyz}}{)}^{2}{]}^{1/2}$$

The highest occupied molecular orbital (HOMO) energy and the lowest unoccupied molecular orbital (LUMO) energy were used to explore the global reactivity parameters (GRPs), such as global hardness (η), global softness (S), global electrophilicity index (ω), electron affinity (EA), ionization potential (IP), electronegativity (X), and the chemical potential (μ) [59,60,61,62,63].

The ionization potential (I) and electronic affinity (A) values are calculated using Equations (7) and (8), respectively.

(7) $$I=-E_{\mathrm{HOMO}}$$

(8) $$A=-E_{\mathrm{LUMO}}$$

Electronegativity (X) and hardness (η) are attained using Equations (9) and (10).

(9) $$X=\frac{I+A}{2}$$

(10) $$\eta =\frac{I-A}{2}$$

To calculate the chemical potential (µ), Equation (11) is used.

(11) $$\mu =\frac{E_{\mathrm{HOMO}}\;+{\;E}_{\mathrm{LUMO}}}{2}$$

The relation between energy variation and maximum electrons transferred can be determined by the magnitude of the electrophilicity (ω), which is calculated according to Equation (12).

(12) $$\omega =\frac{\mu^{2}}{2\eta }$$

For calculating the value of softness (S), Equation (13) is used.

(13) $$\sigma =\frac{1}{2\eta }$$

## 4. Conclusions

This study explores the effect of different substitutions of the π-conjugated linker and diverse acceptor units in carbazole units on NLO properties of **Q3**–**Q5**. We observed differences in NLO behavior, which is strongly dependent on diverse acceptor units, the length of the alkyl chains, and the π-conjugated linker attachment in carbazole to the quinoline skeleton. NBO analysis shows that hyperconjugative interactions, occurring among bonds and intra-molecular charge transfer, are due to electrons’ delocalization. Designed compounds **Q3D1**-**Q3D3**, **Q4D1**, **Q4D2**, and **Q5D1** exhibit strong redshift absorption compared to **Q3**–**Q5**. The decreasing order of softness among all the investigated molecules is **Q3D2** > **Q3D3** > **Q4D2** > **Q4D1** > **Q5D1** > **Q3D1** > **Q4** > **Q3** > **Q5**. The same order exists for bandgaps and there is an inverse pattern for hardness. The designed compounds of **Q4** with -C_8_H_17_ and **Q5** with -C_10_H_21_ moieties exhibited a more promising NLO response than **Q3** with -CH_3_. This difference in NLO efficacy results from the alkyl chain length difference and diverse acceptors. Moreover, the dipole polarizability magnitudes of all designed compounds (**Q3D1**–**Q3D3**), **(Q4D1**, **Q4D2**), and **(Q5D1**) exceed the dipole polarizabilities of corresponding reference compounds (**Q3**, **Q4**, and **Q5**). The second-order polarizability (β_tot_) magnitudes of **Q3D1**, **Q3D2**, and **Q3D3** exceed 2.38, 4.99, and 3.99 times that of **Q3**. The β_tot_ for **Q4D1** and **Q4D2** were 17.55 and 18.10 times that of **Q4**, while β_tot_ for (**Q5D1**) was 16.16 times that of **Q5**. Among the all investigated compounds, the highest value of β_tot_, 59,316.4 (a.u), belongs to **Q4D2**. In short, this data disclosed that entitled compounds might become promising materials in the NLO field. Further, high-performance NLO active entitled compounds could become interesting in synthetic chemistry for lab researchers.

## Figures and Tables

**Table 1:** E_HOMO_, E_LUMO_, and energy gap (E_LUMO_–E_HOMO_) of the investigated compounds in eV using B3LYP/6-311G (d,p) level of theory.

Compounds	E_(__HOMO)_	E_(__LUMO)_	Bandgap (E_LUMO_–E_HOMO_)
**Q3**	-5.593	−1.816	3.777
**Q3D1**	−5.828	−2.781	3.047
**Q3D2**	−5.598	−3.441	2.157
**Q3D3**	−5.535	−3.222	2.313
**Q4**	−5.546	−1.768	3.778
**Q4D1**	−5.522	−2.928	2.594
**Q4D2**	−5.618	−3.126	2.492
**Q5**	−5.540	−1.765	3.775
**Q5D1**	−5.488	−2.879	2.609

H: HOMO, L: LUMO.

**Table 2:** Calculated global reactivity parameters using the energies of HOMO and LUMO.

Compounds	I	A	X	η	μ	ω	S
**Q3**	5.593	1.816	3.704	1.888	−3.704	3.633	0.2647
**Q3D1**	5.828	2.781	4.304	1.523	−4.304	6.080	0.3280
**Q3D2**	5.598	3.441	4.519	1.078	−4.519	9.469	0.4630
**Q3D3**	5.535	3.222	4.378	1.156	−4.378	8.288	0.4320
**Q4**	5.546	1.768	3.655	1.889	−3.657	3.539	0.2646
**Q4D1**	5.522	2.928	4.225	1.297	−4.225	6.881	0.3850
**Q4D2**	5.618	3.126	4.372	1.246	−4.372	7.670	0.4010
**Q5**	5.540	1.765	3.652	1.887	−3.652	3.533	0.2649
**Q5D1**	5.488	2.879	4.183	1.304	−4.183	6.708	0.3830

IP = ionization potential, EA = electron affinity, X = electronegativity, μ = chemical potential, η = global hardness, S = global softness, and ω = global electrophilicity. Units in eV.

**Table 3:** Dipole polarizabilities and major contributing tensors (a.u.) of **Q3**–**Q5D1**.

Systems	α_xx_	α_yy_	α_zz_	<α>
**Q3**	672.576	539.206	221.601	477.794
**Q3D1**	993.607	751.135	324.147	689.629
**Q3D2**	1055.229	741.468	312.972	703.223
**Q3D3**	1019.920	750.156	319.464	696.513
**Q4**	798.885	687.857	419.76	635.500
**Q4D1**	1673.744	875.709	481.294	1010.249
**Q4D2**	1707.951	868.809	482.809	1019.856
**Q5**	730.898	855.859	467.796	684.851
**Q5D1**	1688.74	930.010	526.738	1048.496

**Table 4:** The computed second-order polarizabilities (β_tot_) and major contributing tensors (a.u) of **Q3**–**Q5D1**.

System	β_xxx_	β_xxy_	β_xyy_	β_yyy_	β_xzz_	β_yzz_	β_zzz_	β_total_
**Q3**	−1530.726	1871.795	−474.090	730.504	−51.232	−50.945	−77.808	3277.62
**Q3D1**	7134.938	−602.979	413.750	2310.371	38.201	17.561	−3.128	7811.75
**Q3D2**	15,811.353	−3037.588	490.217	1138.007	−38.279	−15.314	−44.940	16,375.60
**Q3D3**	13,250.577	−884.401	−113.490	1410.887	−51.728	−14.813	−47.341	13,095.90
**Q4**	1303.257	−1614.86	461.9083	−732.367	94.1197	16.408	−3.3530	3000.35
**Q4D1**	−50,840.435	4690.332	−737.360	−762.310	−469.516	105.223	63.545	52,662.1
**Q4D2**	−56,982.727	5969.852	−1009.638	−703.564	−573.947	143.688	56.811	59,316.4
**Q5**	−595.429	−141.705	−1439.05	−1813.59	−43.2138	−228.978	57.493	3103.51
**Q5D1**	−48,440.596	5639.601	−697.923	−541.280	−337.387	120.393	26.346	50,156.00

**Table 5:** Computed transition energy (eV), maximum absorption wavelengths (λ_max_/nm), oscillator strengths (f_os_), light-harvesting efficiency (LHE), transition moment ($M_{X}^{\mathrm{gm}}$ a.u.), and transition natures of analyzed compounds.

Compounds	E_ge_ (eV)	λ_max_ (nm)	ƒ_os_	LHE	∆μ_gm_ (a.u)	Major MO Transitions
**Q3**	3.397	364.97 (349)	1.026	0.905	5.842	H-2→LUMO (92%)
**Q3D1**	2.699	459.35	0.652	0.777	7.088	H-1→LUMO (95%)
**Q3D2**	2.332	531.57	0.707	0.803	7.366	H-1→LUMO (98%)
**Q3D3**	2.445	507.09	0.673	0.788	7.611	H-1→LUMO (98%)
**Q4**	3.435	360.90 (347)	1.005	0.901	3.854	H-2→LUMO (92%)
**Q4D1**	2.087	593.82	1.479	0.966	9.309	HOMO→LUMO (99%)
**Q4D2**	2.019	613.87	1.476	0.966	9.345	HOMO→LUMO (99%)
**Q5**	3.428	361.67 (323)	1.025	0.905	3.658	H-2→LUMO (93%)
**Q5D1**	2.102	589.61	1.454	0.964	7.317	HOMO→LUMO (99%)

## Data Availability

Not applicable.

## Supplementary Materials

The following are available online. Table S1. Cartesian coordinates of **Q3**; Table S2. Cartesian coordinates of **Q3D1**; Table S3: Cartesian coordinates of **Q3D2**; Table S4: Cartesian coordinates of **Q3D3**; Table S5: Cartesian coordinates of **Q4**; Table S6: Cartesian coordinates of **Q4D1**; Table S7: Cartesian coordinates of **Q4D2**; Table S8: Cartesian coordinates of **Q5**; Table S9: Cartesian coordinates of **Q5D1**; Table S10: Second order Perturbation theory analysis of Fock matrix in **Q3**; Table S11: Second order Perturbation theory analysis of Fock matrix in **Q4**; Table S12: Second order Perturbation theory analysis of Fock matrix in **Q5**; Table S13:Second order Perturbation theory analysis of Fock matrix in **Q3D1**; Table S14: Second order Perturbation theory analysis of Fock matrix in **Q3D2**; Table S15: Second order Perturbation theory analysis of Fock matrix in **Q3D3**; Table S16: Second order Perturbation theory analysis of Fock matrix in NBO **Q4D1**; Table S17: Second order Perturbation theory analysis of Fock matrix in **Q4D2**; Table S18: Second order Perturbation theory analysis of Fock matrix in **Q5D1**; Table S19: Wave length, excitation energy and oscillator strength of investigated compound **Q3**. Table S20: Wave length, excitation energy and oscillator strength of investigated compound **Q4**; Table S21: Wave length, excitation energy and oscillator strength of investigated compound **Q5**. Table S22: Wave length, excitation energy and oscillator strength of investigated compound **Q3D1**; Table S23: Wave length, excitation energy and oscillator strength of investigated compound **Q3D2**; Table S25: Wave length, excitation energy and oscillator strength of investigated compound **Q3D3**; Table S26: Wave length, excitation energy and oscillator strength of investigated compound **Q4D1**; Table S27: Wave length, excitation energy and oscillator strength of investigated compound **Q4D2**; Table S28: Wave length, excitation energy and oscillator strength of investigated compound **Q5D1**; Figure S1:Graph of investigated compound **Q3**; Figure S2: Graph of of investigated compound **Q4**; Figure S3: Graph of of investigated compound **Q5**. Figure S4: Graph of investigated compound **Q3D1**; Figure S5:Graph of investigated compound **Q3D2**; Figure S6: Graph of investigated compound **Q3D3**; Figure S7: Graph of investigated compound **Q4D1**; Figure S8:Graph of investigated compound **Q4D2**; Figure S9: Graph of investigated compound **Q5D1**.

## Sample Availability

The original output log files are available from the authors.
