# Supplementary material for: Key Electronic, Linear and Nonlinear Optical Properties of Designed Disubstituted Quinoline with Carbazole Compounds
Source: Molecules. 2021 May 7;26(9):2760. doi: 10.3390/molecules26092760 (PMC8125273; doi:10.3390/molecules26092760)
Supplement: Supplementary file 1 [file molecules-26-02760-s001.zip › molecules-1151657-supplementary.pdf]

Supplementary Information

Key Electronic, Linear and Nonlinear Optical Properties of Designed Disubstituted Quinoline with Carbazole Compounds

Bakhat Ali[1, 2], Muhammad Khalid*[2], Sumreen Asim[2], Muhammad Usman Khan[3], Zahid Iqbal[2], Ajaz Hussain[4], Riaz Hussain[3], Sarfraz Ahmed[5], Akbar Ali[6], Amjad Hussain[3], Muhammad Imran[7], Mohammed A. Assiri [7], Muhammad Fayyaz ur Rehman[6], Chenxi Wang*[8] and Changrui Lu*[1]

[1]Department of Chemistry, Chemical Engineering and Biotechnology, Donghua University, Shanghai, 201620, China, B.A. bakhat.ali@kfueit.edu.pk, C.L. crlu@dhu.edu.cn

[2]Department of Chemistry, Khwaja Fareed University of Engineering & Information Technology, Rahim Yar Khan, 64200, Pakistan M.K. Khalid@iq.usp.br, S.A. sumreen.asim@kfueit.edu.pk, Z.I. mrzahidiqbal77@gmail.com

[3]Department of Chemistry, University of Okara, Okara-56300, Pakistan, M.U.K. usman.chemistry@gmail.com, R.H. riazhussain@uo.edu.pk, A.H. amjadhussain@uo.edu.pk

[4]Institute of Chemical Sciences, Bahauddin Zakariya University, Multan-60800, Pakistan A.H. jaz_hussain01@yahoo.com

[5]KBCMA College of Veterinary and Animal Sciences, Narowal,51600, Pakistan S.A. sarfraz.ahmed@uvas.edu.pk

[6]Institute of Chemistry, University of Sargodha, Sargodha 40100, Pakistan. A.A. akbarchm@gmail.com M.F.R fayyaz9@gmail.com

[7]Department of Chemistry, Faculty of Science, King Khalid University, P.O. Box 9004, Abha 61413, Saudi Arabia. M.I. imranchemist@gmail.com, M.A.A. maassiri@kku.edu.sa

[8] Department of Cardiovascular Surgery, Renji Hospital, School of medicine, Shanghai Jiaotong University, Shanghai, China; C.X.W. wangchenxirj@yeah.net

***Corresponding authors E-mail addresses:**

(C.L.) crlu@dhu.edu.cn; (M.K.)muhammad.khalid@kfueit.edu.pk; Khalid@iq.usp.br; (C.W.) wangchenxirj@yeah.net

Table S1. Cartesian coordinates of **Q3**

Atom	X-axis	Y-axis	Z-axis
C	2.253471	5.298094	0.553167
C	2.324448	3.943818	0.324131
C	1.144773	3.173102	0.162311
C	-0.124	3.825139	0.272675
C	-0.16103	5.225989	0.499894
C	1.000223	5.946158	0.633342
H	3.164055	5.872827	0.67919
C	1.131415	1.762535	-0.08107
H	-1.13603	5.692211	0.5726
H	0.959375	7.01506	0.811086
C	-1.3048	1.857724	-5.3E-05
C	-0.08993	1.130464	-0.14898
H	-0.11724	0.072871	-0.37448
C	-2.62603	1.178061	-0.08174
C	-3.7834	1.95175	-0.3189
C	-2.74296	-0.20766	0.084314
C	-5.03799	1.370662	-0.4004
H	-3.6611	3.02023	-0.42935
C	-4.00642	-0.78762	-0.00367
H	-1.87603	-0.81553	0.307417
C	-5.16904	-0.01236	-0.24192
H	-5.91052	1.987875	-0.58436
N	-1.30849	3.166194	0.185909
N	-4.36916	-2.12397	0.11623
C	-5.74801	-2.22326	-0.01426
C	-6.56942	-3.35025	0.055419
C	-7.93647	-3.16973	-0.12733
C	-8.48131	-1.89947	-0.36929
C	-7.66264	-0.77859	-0.43088
C	-6.28484	-0.92959	-0.25045
H	-6.16665	-4.33668	0.251719
H	-8.59402	-4.03034	-0.07715
H	-9.55127	-1.7948	-0.50593
H	-8.08781	0.201931	-0.6147
C	2.380658	0.975136	-0.27428
C	2.644839	-0.11309	0.562421
C	3.271594	1.290742	-1.32215
C	3.802631	-0.85899	0.342154
H	1.961189	-0.3496	1.36873
C	4.412636	0.533675	-1.54977
H	3.041369	2.124546	-1.97414

C	4.693695	-0.55307	-0.71863
H	5.074505	0.783671	-2.37153
C	5.757707	-1.53017	-0.66095
C	5.464413	-2.38223	0.4356
C	6.907438	-1.75888	-1.42174
C	6.293049	-3.45595	0.766496
C	7.738134	-2.82249	-1.09075
H	7.147325	-1.11601	-2.26147
C	7.430033	-3.66025	-0.00827
H	6.064148	-4.11774	1.593166
H	8.631766	-3.01094	-1.67405
H	8.089247	-4.48761	0.228989
N	4.291004	-1.95277	1.043136
H	3.287431	3.451905	0.274162
C	3.645539	-2.59002	2.170808
H	4.400086	-3.02584	2.826954
H	3.093797	-1.84597	2.746649
H	2.95278	-3.37968	1.857703
C	-3.46556	-3.21552	0.406434
H	-2.50838	-3.04383	-0.08938
H	-3.28996	-3.33166	1.482293
H	-3.87875	-4.14625	0.016698

Table S2. Cartesian coordinates of **Q3D1.**

Atom	X-axis	Y-axis	Z-axis
C	3.60604	7.013511	-0.64312
C	2.672428	6.033345	-0.39968
C	3.063481	4.677194	-0.26163
C	4.446442	4.341373	-0.41551
C	5.384692	5.379071	-0.6567
C	4.975092	6.685001	-0.76364
H	3.29011	8.045087	-0.74975
C	2.157566	3.597069	-0.01294
H	6.424833	5.094944	-0.75961
H	5.700518	7.468709	-0.95144
C	4.063023	2.077526	-0.15165
C	2.672196	2.320614	0.025066
H	1.994999	1.501312	0.219821
C	4.620397	0.696403	-0.11016
C	5.992994	0.492762	-0.37825
C	3.811651	-0.40744	0.184589
C	6.557605	-0.77258	-0.36725
H	6.595631	1.363182	-0.59731
C	4.389457	-1.66918	0.197482
H	2.758444	-0.3043	0.403999
C	5.755977	-1.88378	-0.0805
H	7.612846	-0.89645	-0.58383
N	4.914246	3.067754	-0.35499
N	3.776772	-2.90551	0.460282
C	4.743773	-3.91108	0.333177
C	4.608252	-5.28701	0.499123
C	5.744821	-6.07053	0.324718
C	6.982948	-5.49793	-0.00281
C	7.11054	-4.12326	-0.16208
C	5.984529	-3.31147	0.004276
H	3.654576	-5.73005	0.758689
H	5.670237	-7.14501	0.447371
H	7.848566	-6.13725	-0.13074
H	8.070717	-3.68599	-0.41176
C	0.69804	3.799301	0.204181
C	-0.21578	3.097896	-0.58805
C	0.232476	4.639081	1.239171
C	-1.57853	3.255739	-0.33693
H	0.143152	2.456433	-1.38346
C	-1.12418	4.781419	1.495523

H	0.952728	5.155813	1.86155
C	-2.04921	4.090431	0.709194
H	-1.45821	5.417755	2.307488
C	-3.49181	4.000592	0.69245
C	-3.82784	3.117096	-0.36691
C	-4.50913	4.571666	1.461886
C	-5.15718	2.800081	-0.65767
C	-5.8329	4.26023	1.176079
H	-4.26905	5.248459	2.274373
C	-6.15026	3.384862	0.125309
H	-5.41792	2.126528	-1.46482
H	-6.6302	4.697375	1.765346
H	-7.19027	3.164851	-0.08997
N	-2.66473	2.690439	-0.99041
H	1.624138	6.291418	-0.32206
C	-2.5962	1.712581	-2.05754
H	-3.45455	1.831476	-2.72028
H	-1.69499	1.878095	-2.64819
H	-2.58545	0.687702	-1.67083
C	2.411302	-3.06746	0.705318
C	1.718381	-2.68662	1.822567
S	1.322158	-3.65219	-0.54641
C	0.318066	-2.86321	1.698886
H	2.210452	-2.29088	2.700859
C	-0.0665	-3.38753	0.485762
H	-0.3878	-2.63928	2.487494
C	-1.40904	-3.66936	0.018094
C	-1.83873	-4.67266	-0.83233
S	-2.72262	-2.64138	0.519359
C	-3.22183	-4.6299	-1.05553
H	-1.17907	-5.42409	-1.24535
C	-3.87956	-3.58652	-0.40952
H	-3.75479	-5.33977	-1.67544
C	-5.28887	-3.39833	-0.55969
H	-5.74839	-4.21815	-1.10751
C	-6.18905	-2.42776	-0.2261
C	-7.60083	-2.73504	-0.60194
C	-5.78524	-1.16126	0.421415
O	-7.93015	-3.63443	-1.34372
O	-8.51094	-1.95292	0.013241
H	-9.37541	-2.26651	-0.29068
O	-6.58038	-0.11368	0.140405
H	-6.18422	0.663682	0.569352

O	-4.78547	-1.03854	1.099856

Table S3: Cartesian coordinates of **Q3D2**

Atom	X-axis	Y-axis	Z-axis
C	3.131028	7.056849	0.766233
C	2.213552	6.074672	0.47495
C	2.628149	4.737015	0.24915
C	4.019629	4.421151	0.370215
C	4.941046	5.461288	0.659653
C	4.507943	6.750152	0.848529
H	2.796068	8.072946	0.941369
C	1.73812	3.660417	-0.06835
H	5.98711	5.190941	0.73658
H	5.220363	7.5357	1.074543
C	3.668609	2.170995	0.00623
C	2.276765	2.396142	-0.17391
H	1.63072	1.576171	-0.45743
C	4.237283	0.799505	-0.09384
C	5.608056	0.628736	-0.38322
C	3.430087	-0.32505	0.110318
C	6.172363	-0.63201	-0.49642
H	6.210114	1.517217	-0.51654
C	4.00995	-1.58248	0.010744
H	2.382572	-0.22598	0.359883
C	5.372668	-1.76447	-0.30491
H	7.224702	-0.7363	-0.73571
N	4.511126	3.160199	0.242807
N	3.407464	-2.84495	0.170854
C	4.380416	-3.82996	-0.06242
C	4.260949	-5.21625	-0.01143
C	5.401024	-5.97059	-0.27306
C	6.626924	-5.35948	-0.57253
C	6.739414	-3.97474	-0.6125
C	5.609858	-3.1927	-0.35624
H	3.319202	-5.69341	0.229415
H	5.337809	-7.05219	-0.24071
H	7.495613	-5.97606	-0.77114
H	7.691081	-3.50705	-0.83805
C	0.28119	3.851761	-0.30213
C	-0.63397	3.051941	0.391339
C	-0.18283	4.781193	-1.25804
C	-1.99321	3.193153	0.113673
H	-0.28148	2.355416	1.142136
C	-1.53762	4.92049	-1.52928

H	0.536607	5.374399	-1.80895
C	-2.46285	4.131321	-0.84292
H	-1.86918	5.633516	-2.27603
C	-3.90282	4.010756	-0.86539
C	-4.23916	3.002713	0.075949
C	-4.91781	4.649279	-1.58435
C	-5.56606	2.632604	0.306203
C	-6.23844	4.28374	-1.35843
H	-4.67739	5.42072	-2.30774
C	-6.55366	3.285304	-0.4231
H	-5.82559	1.860879	1.020392
H	-7.0354	4.77147	-1.90752
H	-7.59137	3.014987	-0.26311
N	-3.07253	2.511668	0.651189
H	1.159511	6.31519	0.427413
C	-2.99452	1.513234	1.699151
H	-3.88415	0.88516	1.680986
H	-2.12711	0.871182	1.530384
H	-2.90433	1.972066	2.690289
C	2.05985	-3.04344	0.455673
C	1.373421	-2.61101	1.561351
S	0.973669	-3.77321	-0.71948
C	-0.01512	-2.8681	1.490449
H	1.865438	-2.1345	2.398209
C	-0.40097	-3.50822	0.332602
H	-0.70837	-2.63131	2.286797
C	-1.72837	-3.92458	-0.05448
C	-2.11118	-4.96009	-0.89222
S	-3.11437	-3.04758	0.537067
C	-3.50094	-5.06643	-1.03323
H	-1.40231	-5.62935	-1.36037
C	-4.21896	-4.11143	-0.32143
H	-3.99019	-5.824	-1.63226
C	-5.63759	-4.02579	-0.31715
H	-6.12818	-4.80526	-0.89244
C	-6.47794	-3.12359	0.282327
C	-7.89056	-3.26469	0.137713
C	-6.02544	-2.01105	1.048221
N	-5.65921	-1.10074	1.660352
N	-9.03404	-3.3847	0.019079

Table S4: Cartesian coordinates of **Q3D3**

Atom	X-axis	Y-axis	Z-axis
C	3.992623	6.775399	0.720867
C	2.987973	5.879086	0.440745
C	3.277438	4.507397	0.223534
C	4.634514	4.066388	0.341631
C	5.648562	5.020128	0.619148
C	5.336059	6.34466	0.799641
H	3.75278	7.819114	0.889657
C	2.291177	3.514815	-0.08298
H	6.665874	4.655733	0.693904
H	6.118569	7.063303	1.016437
C	4.077143	1.855089	-0.00451
C	2.711104	2.205833	-0.18174
H	1.992081	1.446373	-0.45759
C	4.517137	0.436543	-0.09696
C	5.866281	0.139824	-0.38684
C	3.610881	-0.60853	0.1143
C	6.313929	-1.16755	-0.4922
H	6.546181	0.969249	-0.52623
C	4.074023	-1.91396	0.020568
H	2.577077	-0.41464	0.366244
C	5.414632	-2.2215	-0.29382
H	7.352474	-1.36841	-0.73039
N	5.00773	2.764833	0.222008
N	3.359654	-3.11414	0.190688
C	4.2375	-4.18508	-0.03501
C	3.990377	-5.55409	0.024605
C	5.056138	-6.41203	-0.23046
C	6.333515	-5.91855	-0.53288
C	6.573022	-4.55045	-0.58335
C	5.520321	-3.66567	-0.33358
H	3.007545	-5.93936	0.266269
H	4.893474	-7.48301	-0.19126
H	7.141721	-6.61405	-0.72625
H	7.563656	-4.17418	-0.8122
C	0.857131	3.838501	-0.31214
C	-0.12459	3.132772	0.392016
C	0.476634	4.800642	-1.27274
C	-1.46634	3.399225	0.12075
H	0.16512	2.410193	1.145058
C	-0.86069	5.064415	-1.53805

H	1.245595	5.320065	-1.83118
C	-1.85169	4.370629	-0.84035
H	-1.12827	5.799813	-2.28882
C	-3.29661	4.385779	-0.8535
C	-3.71983	3.419641	0.097037
C	-4.25197	5.111803	-1.57111
C	-5.07417	3.175428	0.336197
C	-5.59956	4.872729	-1.33535
H	-3.94504	5.852856	-2.30113
C	-6.00063	3.913292	-0.39194
H	-5.40003	2.433048	1.054177
H	-6.3507	5.42966	-1.88337
H	-7.05807	3.741516	-0.22467
N	-2.60096	2.826157	0.669903
H	1.96026	6.214969	0.395008
C	-2.60903	1.835506	1.728701
H	-3.55997	1.304985	1.734814
H	-1.8182	1.102748	1.552813
H	-2.45096	2.293927	2.711512
C	1.996895	-3.18764	0.471873
C	1.357246	-2.74604	1.600557
S	0.843032	-3.74187	-0.73441
C	-0.05117	-2.85799	1.522998
H	1.89448	-2.36275	2.457387
C	-0.49825	-3.39379	0.335214
H	-0.71853	-2.59248	2.332252
C	-1.86214	-3.64931	-0.07056
C	-2.3478	-4.60953	-0.94272
S	-3.14785	-2.64485	0.542067
C	-3.74049	-4.55718	-1.09493
H	-1.7122	-5.33775	-1.42792
C	-4.3549	-3.552	-0.35759
H	-4.30664	-5.2355	-1.72046
C	-5.75522	-3.30571	-0.36007
H	-6.33802	-4.00064	-0.95953
C	-6.50351	-2.34121	0.251583
C	-7.97455	-2.37761	0.03305
C	-5.9488	-1.30882	1.054333
O	-8.54767	-3.20332	-0.63647
O	-8.60817	-1.37418	0.673204
H	-9.55193	-1.47051	0.47877
N	-5.47737	-0.46893	1.695473

Table S5: Cartesian coordinates of **Q4**

Atom	X-axis	Y-axis	Z-axis
C	2.171426	-7.19752	0.724012
C	2.248953	-5.86656	0.387236
C	1.075846	-5.07604	0.282021
C	-0.19024	-5.68174	0.561868
C	-0.23448	-7.05937	0.901177
C	0.918582	-7.80082	0.97667
H	3.077054	-7.78843	0.802021
C	1.07355	-3.68297	-0.04531
H	-1.20808	-7.49123	1.098342
H	0.872473	-8.85217	1.23816
C	-1.34995	-3.71631	0.214318
C	-0.13637	-3.0269	-0.06337
H	-0.15846	-1.96899	-0.29017
C	-2.65534	-3.00524	0.179019
C	-3.72514	-3.48795	0.963217
C	-2.83649	-1.87304	-0.62373
C	-4.95488	-2.85014	0.974264
H	-3.55699	-4.37495	1.558766
C	-4.08113	-1.24626	-0.62406
H	-2.03486	-1.51031	-1.25438
C	-5.15001	-1.71482	0.181113
H	-5.75907	-3.23249	1.593353
N	-1.36587	-5.00072	0.523452
N	-4.50853	-0.13989	-1.35016
C	-5.82841	0.127396	-1.01045
C	-6.67606	1.139102	-1.46735
C	-7.9735	1.175047	-0.96755
C	-8.4239	0.231616	-0.03134
C	-7.57778	-0.77186	0.424309
C	-6.26852	-0.83327	-0.06146
H	-6.34155	1.87968	-2.18411
H	-8.64924	1.951693	-1.30791
H	-9.44091	0.28994	0.338457
H	-7.92889	-1.49906	1.148175
C	2.324349	-2.9311	-0.34549
C	2.680848	-1.84445	0.457488
C	3.12057	-3.27857	-1.45729
C	3.834624	-1.12765	0.138303
H	2.070264	-1.58511	1.313887
C	4.25865	-2.55221	-1.78112

H	2.819733	-4.11268	-2.07981
C	4.630798	-1.46586	-0.98593
H	4.848773	-2.82725	-2.64833
C	5.722998	-0.51926	-1.01893
C	5.540387	0.347145	0.090649
C	6.817489	-0.33211	-1.86754
C	6.430607	1.391201	0.351543
C	7.706552	0.703603	-1.60801
H	6.97131	-0.98664	-2.71846
C	7.511177	1.553749	-0.50886
H	6.290654	2.060023	1.192188
H	8.558887	0.859628	-2.25879
H	8.215609	2.357323	-0.32568
N	4.401614	-0.03988	0.78799
H	3.212429	-5.40709	0.206843
C	3.875601	0.59872	1.984287
H	3.429277	-0.17789	2.612508
H	4.720156	1.001854	2.549706
C	-3.71073	0.604951	-2.31011
H	-3.03443	-0.09851	-2.80479
H	-4.38303	0.979979	-3.08723
C	2.848852	1.70444	1.702147
H	3.314698	2.474301	1.077375
H	2.025795	1.283352	1.114446
C	2.301337	2.332383	2.988323
H	1.857597	1.547878	3.614849
H	3.130943	2.753078	3.571252
C	1.256203	3.425326	2.736588
H	0.429369	3.005002	2.150226
H	1.699067	4.214531	2.115909
C	0.698922	4.044638	4.023369
H	0.262058	3.252546	4.644913
H	1.525141	4.468797	4.608442
C	-0.35441	5.130091	3.775508
H	0.081533	5.924619	3.155982
H	-1.17973	4.706402	3.188809
C	-0.91614	5.745657	5.062254
H	-1.35037	4.95134	5.681326
H	-0.09213	6.170675	5.648279
C	-2.91084	1.764253	-1.69927
H	-3.60314	2.456262	-1.20758
H	-2.25572	1.370594	-0.91428
C	-2.08031	2.509967	-2.74898

H	-1.40199	1.80327	-3.24454
H	-2.74543	2.892125	-3.53442
C	-1.26207	3.672043	-2.17443
H	-0.59613	3.291914	-1.3893
H	-1.93805	4.382586	-1.68209
C	-0.43098	4.409941	-3.23001
H	0.241162	3.696111	-3.72332
H	-1.09843	4.789769	-4.0144
C	0.394338	5.571046	-2.66463
H	1.06274	5.192457	-1.8803
H	-0.27607	6.287331	-2.17198
C	1.225384	6.304825	-3.72323
H	1.894362	5.588667	-4.21549
H	0.557615	6.683746	-4.50653
C	2.049349	7.462505	-3.15275
H	2.75264	7.110274	-2.39156
H	1.40553	8.213845	-2.68473
C	-1.97034	6.826318	4.806017
H	-1.55622	7.651446	4.218104
H	-2.82386	6.422549	4.252675
H	-2.35004	7.244258	5.742306
H	2.62921	7.962793	-3.93313

Table S6: Cartesian coordinates of **Q4D1**

Atom	X-axis	Y-axis	Z-axis
C	7.092458	-6.89589	1.077744
C	7.026412	-5.56154	0.740682
C	5.776794	-4.93848	0.48097
C	4.580665	-5.71863	0.609579
C	4.684595	-7.09362	0.953502
C	5.91273	-7.67093	1.178099
H	8.056422	-7.35565	1.274486
C	5.62095	-3.55398	0.141171
H	3.761168	-7.65741	1.034087
H	5.980577	-8.72228	1.442097
C	3.208334	-3.92793	0.110306
C	4.337757	-3.07221	-0.02449
H	4.19609	-2.02389	-0.26089
C	1.82688	-3.41262	-0.09021
C	0.748562	-4.07416	0.541758
C	1.58135	-2.29459	-0.8994
C	-0.55753	-3.63031	0.394784
H	0.973135	-4.94367	1.147511
C	0.26422	-1.86141	-1.05423
H	2.393827	-1.7975	-1.41751
C	-0.81706	-2.5111	-0.40579
H	-1.36923	-4.14873	0.897178
N	3.33251	-5.20957	0.422395
N	-0.22962	-0.80505	-1.81956
C	-1.60386	-0.74894	-1.65615
C	-2.53458	0.141455	-2.20226
C	-3.87472	-0.03716	-1.88608
C	-4.31587	-1.07523	-1.02866
C	-3.36662	-1.95172	-0.47804
C	-2.0166	-1.79922	-0.78891
H	-2.2326	0.948408	-2.8616
H	-4.60851	0.629468	-2.32873
H	-3.68274	-2.73152	0.20757
C	6.779196	-2.63125	-0.01566
C	6.822541	-1.45089	0.737504
C	7.805293	-2.91207	-0.94737
C	7.900771	-0.58162	0.55722
H	6.036653	-1.23983	1.455149
C	8.867631	-2.03488	-1.13548
H	7.743792	-3.81832	-1.54062

C	8.930079	-0.85783	-0.3826
H	9.63863	-2.26314	-1.86577
C	9.865493	0.243062	-0.30841
C	9.362129	1.135048	0.676384
C	11.06048	0.547854	-0.9702
C	10.03136	2.320175	0.997853
C	11.73137	1.724375	-0.65014
H	11.45836	-0.12527	-1.72429
C	11.21864	2.598133	0.324217
H	9.644509	3.007568	1.743152
H	12.65865	1.97271	-1.15689
H	11.75673	3.512326	0.556975
N	8.181348	0.617701	1.200641
H	7.93342	-4.97099	0.680109
C	7.351356	1.258006	2.208535
H	6.865273	0.471896	2.796442
H	8.012492	1.793007	2.899141
C	0.569919	0.105095	-2.62477
H	1.394641	-0.46789	-3.06296
H	-0.04826	0.441916	-3.46363
C	6.301753	2.21774	1.629135
H	6.812304	2.975904	1.022067
H	5.649546	1.660834	0.944366
C	5.462446	2.897397	2.717349
H	4.961831	2.131639	3.326908
H	6.127367	3.442414	3.402586
C	4.41243	3.864525	2.15664
H	3.73933	3.316968	1.481742
H	4.913149	4.62126	1.536104
C	3.583118	4.565894	3.239239
H	3.081117	3.809936	3.859658
H	4.257153	5.112227	3.914295
C	2.536887	5.534774	2.67506
H	3.038742	6.286224	2.048645
H	1.858665	4.987544	2.004672
C	1.71306	6.246584	3.755104
H	1.210348	5.49597	4.380032
H	2.391147	6.792673	4.424976
C	1.114909	1.312425	-1.8478
H	0.275011	1.851806	-1.39226
H	1.736849	0.951398	-1.01897
C	1.925473	2.259516	-2.74021
H	2.74073	1.700473	-3.22136

H	1.286698	2.624732	-3.55708
C	2.513461	3.457565	-1.98432
H	3.16923	3.091618	-1.18159
H	1.702419	4.007002	-1.48593
C	3.300096	4.418691	-2.88364
H	4.10098	3.864687	-3.39364
H	2.638817	4.793343	-3.67794
C	3.908438	5.607333	-2.12958
H	4.578562	5.233221	-1.34213
H	3.1102	6.156861	-1.61024
C	4.682234	6.574811	-3.03328
H	5.476579	6.024538	-3.55563
H	4.010678	6.952142	-3.8165
C	5.2936	7.755832	-2.27377
H	5.999808	7.413183	-1.50887
H	4.520435	8.345332	-1.768
C	0.672528	7.21336	3.182278
H	1.147613	7.995066	2.578644
H	-0.0434	6.689615	2.538728
H	0.104189	7.707112	3.977
H	5.835155	8.427665	-2.94742
C	-5.73569	-1.24696	-0.71731
C	-6.4036	-2.40084	-0.35386
S	-6.84991	0.105701	-0.75203
C	-7.78416	-2.21455	-0.11973
H	-5.91405	-3.3655	-0.2949
C	-8.20973	-0.91141	-0.30012
H	-8.45787	-3.02065	0.149744
C	-9.53412	-0.3639	-0.16685
C	-9.97701	0.915636	-0.48989
S	-10.8507	-1.31414	0.471975
C	-11.3407	1.130122	-0.23941
H	-9.32255	1.671033	-0.90914
C	-11.9933	0.018334	0.286595
H	-11.8452	2.066101	-0.43935
C	-13.3485	-0.2005	0.674177
H	-13.5799	-1.1916	1.053716
C	-14.4159	0.661436	0.640232
C	-15.7653	0.24932	1.090605
O	-16.7434	0.967533	1.082179
O	-15.81	-1.03876	1.525167
H	-16.7317	-1.19002	1.790094
C	-14.3151	2.006067	0.174369

N	-14.1867	3.096277	-0.21446

T

Table S7: Cartesian coordinates of **Q4D2**

Atom	X-axis	Y-axis	Z-axis
C	6.778989	-6.89273	1.055368
C	6.709137	-5.55863	0.718119
C	5.45699	-4.93696	0.4674
C	4.262657	-5.71828	0.605523
C	4.370459	-7.09297	0.949387
C	5.600828	-7.66892	1.164985
H	7.744844	-7.35142	1.245142
C	5.297397	-3.55283	0.12789
H	3.448254	-7.65766	1.037272
H	5.671737	-8.72005	1.429001
C	2.885164	-3.92916	0.115547
C	4.012475	-3.07238	-0.0286
H	3.868048	-2.02435	-0.26465
C	1.501523	-3.41545	-0.07448
C	0.429275	-4.07643	0.568355
C	1.248099	-2.29969	-0.88425
C	-0.87841	-3.63407	0.431814
H	0.659925	-4.94434	1.174094
C	-0.07071	-1.86806	-1.02846
H	2.055708	-1.80327	-1.41051
C	-1.14563	-2.5169	-0.3691
H	-1.68522	-4.15202	0.942495
N	3.012646	-5.21041	0.427623
N	-0.57211	-0.81361	-1.79211
C	-1.94425	-0.75772	-1.6167
C	-2.88027	0.131176	-2.15661
C	-4.2172	-0.04668	-1.82757
C	-4.65009	-1.08291	-0.96355
C	-3.69566	-1.95863	-0.41994
C	-2.34885	-1.80642	-0.74319
H	-2.5845	0.936404	-2.82079
H	-4.95498	0.618815	-2.2653
H	-4.00503	-2.73703	0.27029
C	6.453546	-2.62904	-0.03764
C	6.500801	-1.44827	0.714692
C	7.473624	-2.90933	-0.97607
C	7.577041	-0.57814	0.526965
H	5.719668	-1.23776	1.437659
C	8.533925	-2.03127	-1.17155
H	7.409045	-3.81584	-1.56861

C	8.600375	-0.85388	-0.41954
H	9.30025	-2.25918	-1.90684
C	9.535403	0.247752	-0.35189
C	9.037846	1.139674	0.635876
C	10.72581	0.553198	-1.02161
C	9.708273	2.325386	0.952623
C	11.39788	1.730316	-0.70632
H	11.11928	-0.11991	-1.77802
C	10.8909	2.604001	0.271119
H	9.325925	3.012679	1.70034
H	12.32165	1.979169	-1.21917
H	11.42987	3.518627	0.500089
N	7.860916	0.621608	1.1681
H	7.615068	-4.96724	0.650473
C	7.036738	1.262377	2.180451
H	6.553567	0.476583	2.771129
H	7.701808	1.797247	2.867377
C	0.220474	0.096143	-2.60506
H	1.041195	-0.47734	-3.04994
H	-0.40502	0.432292	-3.4387
C	5.984525	2.222423	1.606334
H	6.492293	2.979849	0.996029
H	5.328303	1.665482	0.925413
C	5.151481	2.903519	2.698458
H	4.653284	2.138695	3.311162
H	5.820381	3.44849	3.379833
C	4.099549	3.871135	2.142217
H	3.422677	3.323587	1.471066
H	4.597934	4.62685	1.518566
C	3.276077	4.574304	3.228097
H	2.77615	3.819464	3.851549
H	3.9538	5.120567	3.899488
C	2.228299	5.543757	2.667788
H	2.728159	6.294198	2.03858
H	1.54664	4.996635	2.000772
C	1.409818	6.257271	3.750764
H	0.908953	5.507691	4.378417
H	2.091264	6.803198	4.417333
C	0.772025	1.303716	-1.83324
H	-0.06402	1.843624	-1.37123
H	1.400433	0.94291	-1.00923
C	1.575785	2.250149	-2.73248
H	2.387529	1.69085	-3.2192

H	0.930916	2.614679	-3.54486
C	2.169046	3.44877	-1.98164
H	2.830211	3.083395	-1.18309
H	1.36145	3.998671	-1.47817
C	2.949715	4.409161	-2.88691
H	3.747646	3.854908	-3.40124
H	2.2834	4.782739	-3.67752
C	3.562168	5.59876	-2.13772
H	4.236746	5.225645	-1.35361
H	2.766785	6.148774	-1.61453
C	4.330806	6.565316	-3.04678
H	5.122535	6.014642	-3.57266
H	3.654992	6.94151	-3.82689
C	4.945814	7.747416	-2.29193
H	5.656061	7.405891	-1.53027
H	4.175147	8.337392	-1.78292
C	0.367914	7.224702	3.181555
H	0.841313	8.005498	2.57543
H	-0.35119	6.701241	2.541301
H	-0.19663	7.719599	3.978266
H	5.483725	8.41849	-2.96922
C	-6.06608	-1.25323	-0.63733
C	-6.72903	-2.40488	-0.25502
S	-7.18221	0.097282	-0.67575
C	-8.10654	-2.21822	-0.00935
H	-6.2375	-3.36808	-0.19066
C	-8.53604	-0.91703	-0.19946
H	-8.77597	-3.02265	0.275504
C	-9.85872	-0.37094	-0.05885
C	-10.3055	0.907822	-0.38502
S	-11.1693	-1.31954	0.594802
C	-11.666	1.122083	-0.1257
H	-9.6546	1.661566	-0.8125
C	-12.3139	0.011314	0.411272
H	-12.1711	2.057553	-0.32732
C	-13.6639	-0.20318	0.808833
H	-13.8951	-1.19204	1.197036
C	-14.7322	0.665261	0.775202
C	-14.6447	2.009047	0.302055
N	-14.558	3.103571	-0.08734
C	-16.0142	0.227599	1.232297
N	-17.0556	-0.13571	1.606184

Table S8: Cartesian coordinates of **Q5**

Atom	X-axis	Y-axis	Z-axis
C	-7.5345	-2.90762	1.475097
C	-6.24627	-2.86448	0.996061
C	-5.61412	-1.62331	0.727796
C	-6.3328	-0.41355	0.987394
C	-7.66254	-0.49447	1.477393
C	-8.25075	-1.71255	1.712551
H	-8.00373	-3.86376	1.677644
C	-4.27129	-1.49312	0.250949
H	-8.18319	0.438156	1.657542
H	-9.26684	-1.76173	2.088013
C	-4.56441	0.924855	0.344171
C	-3.76676	-0.22411	0.079874
H	-2.74655	-0.10556	-0.26062
C	-4.02758	2.295453	0.132754
C	-4.59086	3.373453	0.849647
C	-2.9868	2.531438	-0.77311
C	-4.12409	4.667555	0.689416
H	-5.40396	3.158982	1.529561
C	-2.53109	3.837383	-0.94272
H	-2.56653	1.720927	-1.35451
C	-3.08312	4.918718	-0.21028
H	-4.56686	5.478549	1.25724
N	-5.80358	0.822468	0.791268
N	-1.54447	4.32136	-1.79522
C	-1.43533	5.693391	-1.60941
C	-0.58133	6.608818	-2.22874
C	-0.68877	7.946911	-1.86488
C	-1.61921	8.372054	-0.90412
C	-2.4648	7.459043	-0.28634
C	-2.37991	6.107542	-0.63306
H	0.146811	6.296289	-2.96771
H	-0.03664	8.675517	-2.3334
H	-1.67531	9.422438	-0.64332
H	-3.18228	7.790919	0.456024
C	-3.41437	-2.67613	-0.04398
C	-2.2313	-2.86554	0.674786
C	-3.77111	-3.57843	-1.06821
C	-1.43009	-3.9649	0.363872
H	-1.96375	-2.17263	1.463457
C	-2.96093	-4.65964	-1.38785

H	-4.68383	-3.40629	-1.62598
C	-1.77891	-4.86808	-0.67319
H	-3.24653	-5.33363	-2.18795
C	-0.73452	-5.86679	-0.72063
C	0.196891	-5.52504	0.294462
C	-0.50934	-6.99829	-1.50956
C	1.340284	-6.29396	0.521766
C	0.626103	-7.76667	-1.28379
H	-1.21216	-7.27396	-2.2881
C	1.538527	-7.41475	-0.27749
H	2.057525	-6.032	1.290343
H	0.812081	-8.64589	-1.8894
H	2.419216	-8.02706	-0.1196
N	-0.24051	-4.37872	0.947732
H	-5.69821	-3.7827	0.826948
C	0.428195	-3.73101	2.065066
H	-0.33975	-3.28583	2.704146
H	0.911663	-4.50835	2.663966
C	-0.74182	3.522665	-2.70674
H	-1.35936	2.692915	-3.06251
H	-0.51836	4.135344	-3.58498
C	1.454492	-2.66655	1.652944
H	2.212575	-3.12974	1.012088
H	0.954554	-1.90577	1.043639
C	2.123101	-2.00939	2.865227
H	1.353467	-1.55312	3.501145
H	2.603273	-2.78278	3.478882
C	3.163139	-0.94608	2.494238
H	2.686643	-0.17189	1.879627
H	3.937873	-1.40128	1.864274
C	3.820929	-0.29102	3.714197
H	3.043727	0.164709	4.340995
H	4.290579	-1.0681	4.331058
C	4.867686	0.770396	3.357636
H	5.647526	0.315477	2.733378
H	4.399949	1.548136	2.740451
C	5.517291	1.421103	4.584363
H	4.736209	1.876361	5.206808
H	5.981208	0.641746	5.202756
C	0.558071	2.98923	-2.0884
H	1.158915	3.835571	-1.73816
H	0.312314	2.395561	-1.20105
C	1.370977	2.142929	-3.07411

H	0.759864	1.299435	-3.42056
H	1.597293	2.740082	-3.96707
C	2.679509	1.60754	-2.48183
H	2.454594	1.014997	-1.5859
H	3.293677	2.450978	-2.14177
C	3.49074	0.75267	-3.46236
H	2.876501	-0.09234	-3.79886
H	3.711811	1.343854	-4.3604
C	4.801559	0.221188	-2.87202
H	4.580357	-0.36627	-1.97161
H	5.417271	1.066555	-2.53875
C	5.611145	-0.63924	-3.8491
H	4.995504	-1.4851	-4.18114
H	5.83175	-0.05263	-4.75028
C	6.922057	-1.16989	-3.25857
H	6.702173	-1.75512	-2.35606
H	7.539536	-0.32443	-2.92808
C	6.567994	2.481721	4.237934
H	7.351316	2.027806	3.616927
H	6.105891	3.262452	3.619838
C	7.731529	-2.03372	-4.23272
H	7.952481	-1.44954	-5.1343
H	7.114925	-2.87859	-4.56269
C	9.03859	-2.56004	-3.63335
H	9.590049	-3.17248	-4.35198
H	9.69298	-1.73787	-3.32702
H	8.848166	-3.17669	-2.74928
C	7.213467	3.130042	5.468152
H	7.674076	2.349665	6.085998
H	6.43068	3.584218	6.087594
C	8.263287	4.187884	5.116553
H	8.702653	4.629614	6.015118
H	9.078009	3.7561	4.526693
H	7.824946	4.999998	4.528002

Table S9: Cartesian coordinates of **Q5D1**

Atom	X-axis	Y-axis	Z-axis
C	6.204893	-7.81671	1.290686
C	6.26875	-6.48824	0.930538
C	5.088688	-5.76368	0.614911
C	3.824669	-6.4339	0.710649
C	3.794933	-7.80613	1.078902
C	4.95878	-8.48389	1.358524
H	7.116898	-8.35518	1.530791
C	5.066964	-4.3775	0.248774
H	2.823381	-8.28587	1.133579
H	4.924589	-9.53202	1.641173
C	2.633331	-4.53957	0.135043
C	3.837783	-3.78882	0.028179
H	3.797608	-2.73663	-0.22914
C	1.310679	-3.90708	-0.11885
C	0.158641	-4.45634	0.490059
C	1.192149	-2.78619	-0.95235
C	-1.09631	-3.89686	0.298384
H	0.285152	-5.33194	1.115194
C	-0.07463	-2.2375	-1.15324
H	2.061309	-2.37457	-1.45307
C	-1.22882	-2.77293	-0.52623
H	-1.96606	-4.32907	0.784875
N	2.633333	-5.82122	0.470435
N	-0.44765	-1.15446	-1.94921
C	-1.81448	-0.96817	-1.82537
C	-2.64321	-0.00479	-2.41042
C	-4.00144	-0.0498	-2.12544
C	-4.55981	-1.02502	-1.26249
C	-3.71168	-1.97801	-0.67512
C	-2.34617	-1.95942	-0.95359
H	-2.25029	0.757126	-3.07535
H	-4.6578	0.675109	-2.59722
H	-4.11653	-2.71138	0.014931
C	6.304849	-3.55954	0.120485
C	6.422477	-2.37499	0.858904
C	7.332248	-3.93796	-0.77434
C	7.571731	-1.59796	0.698365
H	5.635513	-2.0901	1.54924
C	8.467115	-3.15219	-0.94261
H	7.214726	-4.84557	-1.35697

C	8.601967	-1.97053	-0.20633
H	9.238125	-3.45407	-1.64569
C	9.618383	-0.94464	-0.12161
C	9.159078	0.001163	0.834297
C	10.85179	-0.7455	-0.75266
C	9.910057	1.135778	1.157044
C	11.60355	0.380691	-0.43131
H	11.21652	-1.46088	-1.48428
C	11.13384	1.308599	0.514194
H	9.558466	1.864267	1.880565
H	12.56131	0.546984	-0.91438
H	11.73532	2.181927	0.748741
N	7.927107	-0.41315	1.331486
H	7.225774	-5.98026	0.894733
C	7.124942	0.298956	2.312982
H	6.589401	-0.44311	2.915266
H	7.808859	0.813115	2.996835
C	0.454651	-0.33476	-2.74276
H	1.247051	-0.98454	-3.13025
H	-0.09887	0.026689	-3.61595
C	6.135117	1.301013	1.700376
H	6.690728	2.016318	1.081006
H	5.458938	0.76445	1.022692
C	5.32543	2.047966	2.766423
H	4.789591	1.321998	3.394413
H	6.013191	2.580058	3.439136
C	4.320229	3.046488	2.178752
H	3.624839	2.512215	1.515827
H	4.854065	3.765926	1.541668
C	3.520883	3.807827	3.243065
H	2.99013	3.087917	3.882045
H	4.217069	4.343214	3.904219
C	2.512859	4.803629	2.655881
H	3.043442	5.520184	2.012782
H	1.814271	4.267145	1.998075
C	1.718574	5.570109	3.720319
H	1.188817	4.853167	4.363612
H	2.417838	6.106302	4.377698
C	1.064096	0.846181	-1.97305
H	0.253562	1.479745	-1.59147
H	1.595936	0.461404	-1.09379
C	2.017704	1.676569	-2.84011
H	2.814373	1.026966	-3.22989

H	1.477369	2.053432	-3.72027
C	2.649632	2.856496	-2.09128
H	3.184881	2.479118	-1.2085
H	1.854626	3.51056	-1.70642
C	3.613743	3.680494	-2.95346
H	4.405542	3.023153	-3.33977
H	3.078538	4.059933	-3.83552
C	4.252751	4.856297	-2.20437
H	4.782244	4.476066	-1.31894
H	3.461569	5.517055	-1.82235
C	5.225502	5.673773	-3.06303
H	6.015599	5.011603	-3.4448
H	4.696315	6.054186	-3.94838
C	5.867245	6.847971	-2.31414
H	6.393865	6.468443	-1.42661
H	5.077939	7.513072	-1.93514
C	0.709917	6.565987	3.134827
H	1.238805	7.282459	2.489856
H	0.008565	6.030162	2.479124
C	6.844888	7.66213	-3.17048
H	6.319346	8.040742	-4.05769
H	7.634307	6.997775	-3.54744
C	7.480434	8.833099	-2.41502
H	8.175239	9.390971	-3.05109
H	6.718426	9.535893	-2.05924
H	8.039344	8.482916	-1.53969
C	-0.08228	7.334959	4.199098
H	0.618427	7.870593	4.854019
H	-0.61092	6.619231	4.8433
C	-1.08725	8.327124	3.606235
H	-1.63512	8.859593	4.390402
H	-0.58465	9.076848	2.984587
H	-1.82346	7.816265	2.975288
C	-5.99568	-1.05194	-0.98016
C	-6.7812	-2.12732	-0.61066
S	-6.97022	0.402949	-1.06094
C	-8.14041	-1.80218	-0.40659
H	-6.39082	-3.13418	-0.52401
C	-8.43179	-0.46668	-0.61693
H	-8.89418	-2.53255	-0.1335
C	-9.69775	0.20941	-0.51388
C	-9.99827	1.529691	-0.83442
S	-11.114	-0.62772	0.082211

C	-11.3414	1.859475	-0.61016
H	-9.26358	2.22318	-1.22619
C	-12.1124	0.810719	-0.11359
H	-11.7645	2.839121	-0.80446
C	-13.4998	0.92147	0.181678
C	-14.3937	-0.00086	0.66233
H	-13.9113	1.908622	-0.00973
C	-14.0322	-1.34662	0.969199
N	-13.7092	-2.43917	1.21135
C	-15.8149	0.341536	0.897993
O	-16.6508	-0.43013	1.320217
O	-16.1074	1.634627	0.590116
H	-17.0534	1.736654	0.783538

Table S10: Second order Perturbation theory analysis of Fock matrix in **Q3**

**Donor (i)**	**Type**	**Acceptor(j)**	**Type**	**E(2)[a][kJ/mol]**	**E(j)-E(i)[b][a.u.]**	**F(i,j)c[a.u.]**
C_1-C_2	π	C_3-C_4	π^*	22.92	0.37	0.086
C_1-C_2	π	C_5-C_6	π^*	25.20	0.39	0.089
C_1-H_7	σ	C_2-C_3	σ^*	5.15	1.19	0.070
C_3-C_4	π	C_8-C_{12}	π^*	25.46	0.36	0.091
C_5-C_6	π	C_3-C_4	π^*	24.72	0.37	0.089
C_8-C_{12}	π	C_{11}-C_{23}	π^*	34.82	0.37	0.103
C_{12}-H_{13}	σ	C_3-C_8	σ^*	5.42	1.19	0.072
C_{15}-C_{17}	σ	C_{21}-C_{30}	σ	5.22	1.36	0.075
C_{15}-C_{17}	π	C_{14}-C_{16}	π^*	30.09	0.36	0.095
C_{17}-C_{21}	σ	C_{21}-C_{30}	σ^*	6.04	1.36	0.081
C_{25}-C_{30}	π	C_{28}-C_{29}	π^*	31.67	0.36	0.097
C_{28}-C_{29}	π	C_{26}-C_{27}	π^*	31.50	0.37	0.097
C_{37}-C_{40}	π	C_{35}-C_{36}	π^*	31.18	0.37	0.097
C_{38}-C_{42}	π	C_{37}-C_{40}	π^*	31.65	0.36	0.097
C_{44}-C_{45}	π	C_{46}-C_{48}	π^*	31.63	0.36	0.097
C_{46}-C_{48}	π	C_{47}-C_{50}	π^*	31.49	0.37	0.097
N_{23}	LP(1)	C_3-C_4	σ^*	11.40	0.99	0.096
N_{23}	LP(1)	C_{11}-C_{12}	σ^*	11.48	0.99	0.096
N_{24}	LP(1)	C_{19}-C_{21}	π^*	47.38	0.36	0.120
N_{24}	LP(1)	C_{25}-C_{30}	π^*	48.10	0.36	0.121
N_{24}	LP(1)	C_{60}-H_{62}	σ^*	7.87	0.75	0.075
N_{54}	LP(1)	C_{38}-C_{42}	π^*	48.44	0.36	0.121
N_{54}	LP(1)	C_{44}-C_{45}	π^*	47.67	0.36	0.120
C_{25}-C_{30}	π	C_{26}-C_{27}	π^*	431.28	0.01	0.095
C_{44}-C_{45}	π	C_{47}-C_{50}	π^*	443.80	0.01	0.095

Table S11: Second order Perturbation theory analysis of Fock matrix in **Q4**

**Donor (i)**	**Type**	**Acceptor(j)**	**Type**	**E(2)[a][kJ/mol]**	**E(j)–E(i)[b][a.u.]**	**F(i,j)c[a.u.]**
C_1-C_2	π	C_3-C_4	π^*	22.94	0.37	0.086
C_1-C_2	π	C_5-C_6	π^*	25.18	0.39	0.089
C_2-C_3	σ	C_3-C_8	σ^*	5.04	1.35	0.074
C_3-C_4	π	C_1-C_2	π^*	23.40	0.36	0.087
C_3-C_4	π	C_5-C_6	π^*	20.18	0.36	0.081
C_3-C_4	π	C_8-C_{12}	π^*	25.52	0.36	0.091
C_5-C_6	π	C_1-C_2	π	24.41	0.39	0.087
C_5-C_6	π	C_3-C_4	π^*	24.72	0.37	0.089
C_3-C_4	π	C_{11}-N_{23}	π^*	19.90	0.34	0.076
C_8-C_{12}	π	C_3-C_4	π^*	18.59	0.37	0.077
C_8-C_{12}	π	C_{11}-N_{23}	π^*	35.04	0.37	0.103
C_{11}-N_{23}	π	C_3-C_4	π^*	27.96	0.42	0.102
C_{11}-N_{23}	π	C_8-C_{12}	π^*	14.22	0.44	0.071
C_{15}-C_{17}	π	C_{14}-C_{16}	π^*	30.30	0.36	0.096
C_{25}-C_{30}	π	C_{28}-C_{29}	π^*	31.69	0.36	0.097
C_{37}-C_{40}	π	C_{35}-C_{36}	π^*	31.32	0.37	0.097
C_{38}-C_{42}	σ	C_{36}-C_{38}	σ^*	5.30	1.37	0.076
C_{40}-C_{42}	σ	C_{42}-C_{44}	σ^*	6.03	1.36	0.081
C_{44}-C_{45}	π	C_{46}-C_{48}	π^*	31.66	0.36	0.097
C_{46}-C_{48}	π	C_{47}-C_{50}	π^*	31.44	0.37	0.097
C_{47}-C_{50}	σ	C_{45}-N_{54}	σ^*	6.62	1.28	0.082
C_{47}-C_{50}	π	C_{44}-C_{45}	π^*	29.34	0.36	0.096
N_{23}	LP(1)	C_3-C_4	σ^*	11.37	0.99	0.125
N_{23}	LP(1)	C_{11}-C_{12}	σ^*	11.58	0.99	0.125
N_{24}	LP(1)	C_{19}-C_{21}	π^*	47.30	0.36	0.024
N_{24}	LP(1)	C_{25}-C_{30}	π^*	47.92	0.36	0.125
N_{54}	LP(1)	C_{38}-C_{42}	π^*	48.35	0.36	0.024
N_{54}	LP(1)	C_{44}-C_{45}	π^*	37.48	0.36	0.125
C_{14}-C_{16}	π	C_{15}-C_{17}	π^*	361.61	0.01	0.095
C_{19}-C_{21}	π	C_{15}-C_{17}	π^*	235.34	0.02	0.095
C_{25}-C_{30}	π	C_{26}-C_{27}	π^*	445.49	0.01	0.095
C_{25}-C_{30}	π	C_{28}-C_{29}	π^*	300.26	0.02	0.096
C_{35}-C_{36}	π	C_8-C_{12}	π^*	39.57	0.01	0.035
C_{38}-C_{42}	π	C_{37}-C_{40}	π^*	326.49	0.01	0.096
C_{44}-C_{45}	π	C_{46}-C_{48}	π^*	304.50	0.01	0.096
C_{44}-C_{45}	π	C_{47}-C_{50}	π^*	449.91	0.01	0.095

Table S12: Second order Perturbation theory analysis of Fock matrix in **Q5**

**Donor (i)**	**Type**	**Acceptor(j)**	**Type**	**E(2)^{a}[kJ/mol]**	**E(j)-E(i)^{b}[a.u.]**	**F(i,j)c[a.u.]**
C_1-C_2	π	C_3-C_4	π^*	22.94	0.37	0.086
C_1-C_2	π	C_5-C_6	π^*	25.18	0.39	0.089
C_1-H_7	σ	C_2-C_3	σ^*	5.14	1.19	0.070
C_2-C_3	σ	C_3-C_8	σ^*	5.05	1.35	0.074
C_3-C_4	π	C_8-C_{12}	π^*	25.51	0.36	0.091
C_3-C_4	π	C_{11}-N_{23}	π^*	19.95	0.34	0.076
C_5-H_9	σ	C_3-C_4	σ^*	5.01	1.17	0.069
C_8-C_{12}	π	C_{11}-N_{23}	π^*	34.93	0.37	0.103
C_{11}-N_{23}	π	C_3-C_4	π^*	27.99	0.42	0.102
C_{11}-N_{23}	π	C_8-C_{12}	π^*	14.21	0.44	0.071
C_{12}-H_{13}	σ	C_3-C_8	σ^*	5.39	1.19	0.072
C_{37}-C_{40}	π	C_{35}-C_{36}	π^*	31.36	0.37	0.097
C_{38}-C_{42}	π	C_{37}-C_{40}	π^*	31.83	0.36	0.098
C_{44}-C_{45}	π	C_{46}-C_{48}	π^*	31.64	0.36	0.097
C_{46}-C_{48}	π	C_{47}-C_{50}	π^*	31.43	0.37	0.097
N_{23}	LP(1)	C_3-C_4	σ^*	11.38	0.99	0.096
N_{23}	LP(1)	C_{11}-C_{12}	σ^*	11.55	0.99	0.043
N_{24}	LP(1)	C_{19}-C_{21}	π^*	47.39	0.36	0.097
N_{24}	LP(1)	C_{25}-C_{30}	π^*	47.94	0.36	0.119
N_{24}	LP(1)	C_{59}-C_{80}	σ^*	7.00	0.74	0.120
N_{54}	LP(1)	C_{38}-C_{42}	π^*	48.35	0.36	0.070
N_{54}	LP(1)	C_{44}-C_{45}	π^*	47.47	0.36	0.120
C_{14}-C_{16}	π	C_{15}-C_{17}	π^*	347.59	0.01	0.095
C_{19}-C_{21}	π	C_{15}-C_{17}	π^*	230.71	0.02	0.095
C_{25}-C_{30}	π	C_{26}-C_{27}	π^*	445.23	0.01	0.095
C_{25}-C_{30}	π	C_{28}-C_{29}	π^*	299.84	0.02	0.096
C_{38}-C_{42}	π	C_{37}-C_{40}	π^*	323.57	0.01	0.096
C_{44}-C_{45}	π	C_{46}-C_{48}	π^*	306.70	0.01	0.096

Table S13: Second order Perturbation theory analysis of Fock matrix in **Q3D1**

**Donor(i)**	**Type**	**Acceptor(j)**	**Type**	**E(2)**	**E(J)E(i)[b](a.u)**	**F(I,j)[c](a.u)**
C1-C2	π	C5-C6	π^*	25.13	0.39	0.089
C2-C3	∂	C3-C8	∂^*	5.04	1.35	0.074
C2-H55	∂	C3-C4	∂^*	4.98	1.17	0.069
C5-C6	π	C1-C2	π^*	24.49	0.39	0.087
C5-H9	∂	C3-C4	∂^*	5.01	1.17	0.069
C8-C12	π	C11-N23	π^*	34.90	0.37	0.103
C8-C12	π	C35-C36	π^*	6.01	0.38	0.044
C11-N23	π	C8-C12	π^*	14.27	0.44	0.071
C11-N23	π	C14-C15	π^*	8.24	0.43	0.056
C12-H13	∂	C3-C8	∂^*	5.42	1.19	0.072
C14-C15	π	C11-N23	π^*	19.50	0.34	0.073
C14-C15	π	C16-C19	π^*	28.86	0.35	0.090
C14-C15	π	C17-C21	π^*	33.96	0.35	0.098
C14-C16	∂	C16-C19	∂^*	5.18	1.41	0.077
C16-C19	π	C14-C15	π^*	31.44	0.37	0.098
C16-C19	π	C17-C21	π^*	25.44	0.37	0.088
C17-C21	π	C14-C15	π^*	31.17	0.35	0.095
C17-C21	π	C16-C19	π^*	31.07	0.35	0.094
C17-C21	π	C29-C30	π^*	19.78	0.36	0.076
C19-C21	∂	N24-C60	∂^*	5.14	1.22	0.071
C25-C26	π	C27-C28	π^*	33.85	0.37	0.100
C25-C26	π	C29-C30	π^*	25.13	0.37	0.087
C26-C27	∂	N24-C25	∂^*	6.88	1.26	0.083
C27-C28	π	C25-C26	π^*	27.68	0.35	0.089
C27-C28	π	C29-C30	π^*	33.63	0.36	0.100
C28-C29	∂	C21-C30	∂^*	5.35	1.35	0.076
C29-C30	π	C17-C21	π^*	20.41	0.36	0.077
C29-C30	π	C25-C26	π^*	30.91	0.35	0.094
C29-C30	π	C27-C28	π^*	29.14	0.36	0.091
C35-C36	π	C37-C40	π^*	23.78	0.38	0.085
C37-C40	π	C35-C36	π^*	31.07	0.37	0.097
C44-C46	π	C45-C47	π^*	30.83	0.35	0.09
C44-C46	π	C48-C50	π^*	29.08	0.35	0.091
C27-C28	π	C29-C30	π^*	33.63	0.36	0.100
C45-C47	π	C44-C46	π^*	24.19	0.37	0.085
C45-C47	π	C48-C50	π^*	35.02	0.36	0.101
C47-C50	∂	C45-N54	∂^*	6.55	1.29	0.082
C48-C50	π	C44-C46	π^*	33.28	0.36	0.100
C48-C50	π	C45-C47	π^*	26.74	0.35	0.088
C60-C61	π	C63-C65	π^*	19.86	0.40	0.083
C63-C65	π	C60-C61	π^*	22.38	0.39	0.086
C63-C65	π	C67-C68	π^*	13.40	0.39	0.067
C67-C68	π	C63-C65	π^*	12.36	0.38	0.063
C67-C68	π	C70-C72	π^*	29.39	0.37	0.097
C70-C72	π	C67-C68	π^*	20.30	0.37	0.080
C70-C72	π	C74-C76	π^*	33.10	0.40	0.104
C74-H75	∂	S69-C72	∂^*	10.29	0.84	0.083
C74-C76	π	C70-C72	π^*	10.86	0.38	0.060
C74-C76	π	C77-O79	π^*	26.68	0.38	0.092

C74-C76	π	C78-O84	π^*	24.90	0.38	0.089
C3	LP(1)	C1-C2	π^*	64.13	0.20	0.125
C3	LP(1)	C8-C12	π^*	65.30	0.20	0.127
N23	LP(1)	C3-C4	∂^*	11.40	0.99	0.096
N23	LP(1)	C11-C12	∂^*	11.39	0.99	0.096
N24	LP(1)	C16-C19	π^*	43.40	0.38	0.116
N24	LP(1)	C25-C26	π^*	44.71	0.38	0.118
N24	LP(1)	C60-S62	∂^*	14.75	0.56	0.087
C42	LP(1)	C44-C46	∂^*	67.53	0.18	0.115
N54	LP(1)	C45-C47	∂^*	52.97	0.36	0.125
S62	LP(2)	C60-C61	∂^*	30.62	0.35	0.093
S62	LP(2)	C63-C65	∂^*	31.45	0.35	0.095
S69	LP(2)	C67-C68	∂^*	35.87	0.34	0.099
S69	LP(2)	C70-C72	∂^*	30.55	0.33	0.090
O79	LP(2)	C76-C77	∂^*	21.28	0.80	0.119
O79	LP(2)	C77-O80	∂^*	36.28	0.75	0.149
O80	LP(2)	C77-O79	π^*	58.85	0.44	0.147
O80	LP(2)	C78-O84	π^*	59.23	0.44	0.147
O84	LP(2)	C76-C78	∂^*	18.31	0.82	0.112
O84	LP(2)	C78-O82	∂^*	35.69	0.76	0.149

Table S14: Second order Perturbation theory analysis of Fock matrix in **Q3D2**

**Donor(i)**	**Type**	**Acceptor(j)**	**Type**	**E(2)**	**E(J)E(i)^b(a.u)**	**F(I,j)^e(a.u)**
N54	LP(1)	C38-C42	π^*	49.19	0.36	0.122
N54	LP(1)	C44-C45	π^*	47.78	0.36	0.12
N24	LP(1)	C25-C30	π^*	40.05	0.37	0.113
N24	LP(1)	C19-C21	π^*	39.04	0.37	0.111
C70-C72	π	C74-C76	π^*	38.41	0.37	0.108
C8-C12	π	C11-N23	π^*	35.4	0.37	0.103
S69	LP(2)	C67-C68	π^*	35.22	0.34	0.099
C38-C42	π	C37-C40	π^*	31.75	0.36	0.098
C44-C45	π	C46-C48	π^*	31.53	0.36	0.097
C28-C29	π	C26-C27	π^*	31.49	0.37	0.096
C46-C48	π	C47-C50	π^*	31.22	0.37	0.097
S62	LP(2)	C60-C61	π^*	31.19	0.34	0.094
C67-C68	π	C70-C72	π^*	31	0.37	0.099
C37-C40	π	C35-C36	π^*	30.98	0.37	0.097
S62	LP(2)	C63-C65	π^*	30.98	0.35	0.093
C26-C27	π	C25-C30	π^*	30.67	0.36	0.097
C14-C16	π	C19-C21	π^*	30.31	0.36	0.096
C25-C30	π	C28-C29	π^*	30.17	0.37	0.096
C15-C17	π	C14-C16	π^*	29.96	0.37	0.095
S69	LP(2)	C70-C72	π^*	29.53	0.34	0.089
C47-C50	π	C44-C45	π^*	29.28	0.36	0.096
C19-C21	π	C14-C16	π^*	28.86	0.36	0.092
C35-C36	π	C38-C42	π^*	28.84	0.36	0.095
C38-C42	π	C35-C36	π^*	28.11	0.35	0.09
C19-C21	π	C15-C17	π^*	28.1	0.37	0.094
C11-N23	π	C3-C4	π^*	27.89	0.42	0.102
C74-C76	π	C77-N80	π^*	27.13	0.5	0.106
C25-C30	π	C26-C27	π^*	26.89	0.36	0.089
C74-C76	π	C78-N79	π^*	26.65	0.49	0.105
C44-C45	π	C47-C50	π^*	26.16	0.36	0.088
C15-C17	π	C19-C21	π^*	25.92	0.36	0.089
C28-C29	π	C25-C30	π^*	25.89	0.35	0.089
C26-C27	π	C28-C29	π^*	25.74	0.37	0.088
C3-C4	π	C8-C12	π^*	25.41	0.36	0.091
C1-C2	π	C5-C6	π^*	25.15	0.39	0.089
C5-C6	π	C3-C4	π^*	24.83	0.36	0.089
C47-C50	π	C46-C48	π^*	24.68	0.37	0.087
C5-C6	π	C1-C2	π^*	24.46	0.39	0.087
C14-C16	π	C15-C17	π^*	24.36	0.38	0.086
C46-C48	π	C44-C45	π^*	24.2	0.36	0.087

C37-C40	π	C38-C42	π^*	24.02	0.36	0.087
C35-C36	π	C37-C40	π^*	23.88	0.37	0.085
C44-C45	π	C38-C42	π^*	23.52	0.34	0.081
C3-C4	π	C1-C2	π^*	23.46	0.36	0.087
C1-C2	π	C3-C4	π^*	22.97	0.36	0.086
C38-C42	π	C44-C45	π^*	22.89	0.34	0.08
C25-C30	π	C19-C21	π^*	22.63	0.35	0.081
C19-C21	π	C25-C30	π^*	22.07	0.35	0.08
C63-C65	π	C60-C61	π^*	21.64	0.39	0.084
C60-C61	π	C63-C65	π^*	21.48	0.4	0.086
C3-C4	π	C5-C6	π^*	20.13	0.37	0.081
C3-C4	π	C11-N23	π^*	19.96	0.34	0.075
C70-C72	π	C67-C68	π^*	19.8	0.37	0.078
C14-C16	π	C11-N23	π^*	18.54	0.35	0.073
C8-C12	π	C3-C4	π^*	18.53	0.37	0.077
C63-C65	π	C67-C68	π^*	16.54	0.38	0.074
C11-N23	π	C8-C12	π^*	14.22	0.44	0.071
C67-C68	π	C63-C65	π^*	13.47	0.39	0.066
N24	LP(1)	C60-S62	∂^*	13.16	0.56	0.083
N80	LP(1)	C76-C77	∂^*	11.89	1.14	0.104
N23	LP(1)	C11-C12	∂^*	11.55	1	0.097
N79	LP(1)	C76-C78	∂^*	11.48	1.14	0.102
N23	LP(1)	C3-C4	∂^*	11.32	0.99	0.095
C74-C76	π	C70-C72	π^*	10.7	0.39	0.061
C76-C78	∂	C78-N79	∂^*	9.64	1.78	0.117
C11-N23	π	C14-C16	π^*	9.53	0.43	0.059
C76-C77	∂	C77-N80	∂^*	9.53	1.78	0.117
N24	LP(1)	C60-C61	π^*	9.51	0.36	0.052
C74-H75	∂	S69-C72	∂^*	9.31	0.86	0.08
C77-N80	∂	C76-C77	∂^*	8.61	1.69	0.108
C78-N79	∂	C76-C78	∂^*	8.49	1.7	0.108
C36-H39	∂	C38-N54	∂^*	0.51	1.11	0.021

Table S15: Second order Perturbation theory analysis of Fock matrix in **Q3D3**

**Donor(i)**	**Type**	**Acceptor(j)**	**Type**	**E(2)**	**E(J)E(i)^{b}(a.u)**	**F(I,j)^{e}(a.u)**
O80	LP(2)	C77-O79	π*	45.47	0.34	0.114
N54	LP(1)	C38-C42	π*	38.11	0.28	0.095
N54	LP(1)	C44-C45	π*	36.78	0.29	0.094
N24	LP(1)	C25-C30	π*	31.59	0.3	0.089
O79	LP(2)	C77-O80	∂*	31.41	0.62	0.127
N24	LP(1)	C19-C21	π*	30.59	0.3	0.088
C70-C72	π	C74-C76	π*	25.75	0.3	0.079
C8-C12	π	C11-N23	π*	24.89	0.28	0.076
S69	LP(2)	C67-C68	π*	24.7	0.26	0.072
C14-C16	π	C19-C21	π*	22.29	0.28	0.073
C74-C76	π	C77-O79	π*	22.23	0.3	0.073
S62	LP(2)	C63-C65	π*	21.94	0.27	0.069
S62	LP(2)	C60-C61	π*	21.62	0.26	0.068
C38-C42	π	C37-C40	π*	21.52	0.28	0.071
C74-C76	π	C78-N82	π*	21.49	0.39	0.084
C11-N23	π	C3-C4	π*	21.28	0.33	0.079
C44-C45	π	C46-C48	π*	21.27	0.28	0.071
S69	LP(2)	C70-C72	π*	21.27	0.26	0.066
C26-C27	π	C25-C30	π*	21.26	0.28	0.072
C28-C29	π	C26-C27	π*	21.23	0.28	0.069
C37-C40	π	C35-C36	π*	21.08	0.28	0.07
C46-C48	π	C47-C50	π*	20.92	0.28	0.069
C47-C50	π	C44-C45	π*	20.87	0.28	0.072
C35-C36	π	C38-C42	π*	20.76	0.29	0.072
C15-C17	π	C14-C16	π*	20.46	0.28	0.069
C25-C30	π	C28-C29	π*	20.34	0.28	0.069
C67-C68	π	C70-C72	π*	19.97	0.29	0.07
O79	LP(2)	C76-C77	∂*	19.67	0.67	0.105
C44-C45	π	C38-C42	π*	19.28	0.27	0.066
C38-C42	π	C35-C36	π*	19.16	0.27	0.065
C19-C21	π	C14-C16	π*	19.14	0.29	0.066
C15-C17	π	C19-C21	π*	19.08	0.28	0.068
C3-C4	π	C8-C12	π*	18.89	0.28	0.069
C25-C30	π	C26-C27	π*	18.83	0.28	0.066
C38-C42	π	C44-C45	π*	18.6	0.28	0.065
C19-C21	π	C15-C17	π*	18.58	0.29	0.068
C28-C29	π	C25-C30	π*	18.57	0.28	0.067
C19-C21	π	C25-C30	π*	18.41	0.28	0.065
C25-C30	π	C19-C21	π*	18.27	0.28	0.065
C44-C45	π	C47-C50	π*	17.99	0.28	0.064
C26-C27	π	C28-C29	π*	17.72	0.29	0.064

C5-C6	π	C1-C2	π*	17.71	0.29	0.065
C1-C2	π	C5-C6	π*	17.54	0.3	0.065
C46-C48	π	C44-C45	π*	17.54	0.28	0.066
C3-C4	π	C1-C2	π*	17.5	0.28	0.066
C5-C6	π	C3-C4	π*	17.5	0.29	0.066
C47-C50	π	C46-C48	π*	17.41	0.29	0.064
C37-C40	π	C38-C42	π*	17.3	0.28	0.066
C14-C16	π	C15-C17	π*	16.88	0.29	0.063
C35-C36	π	C37-C40	π*	16.63	0.29	0.062
C1-C2	π	C3-C4	π*	16.28	0.29	0.064
C63-C65	π	C60-C61	π*	15.8	0.29	0.063
C14-C16	π	C11-N23	π*	15.61	0.28	0.059
C70-C72	π	C67-C68	π*	15.18	0.29	0.06
C60-C61	π	C63-C65	π*	15.14	0.3	0.063
C3-C4	π	C5-C6	π*	14.95	0.28	0.062
C3-C4	π	C11-N23	π*	14.25	0.26	0.056
C8-C12	π	C3-C4	π*	13.68	0.29	0.059
C63-C65	π	C67-C68	π*	13.18	0.29	0.058
N24	LP(1)	C60-S62	∂*	12.55	0.45	0.072
C67-C68	π	C63-C65	π*	11.8	0.3	0.054
N82	LP(1)	C76-C78	∂*	11.49	1.02	0.097
C74-C76	π	C70-C72	π*	11.03	0.29	0.053
C11-N23	π	C8-C12	π*	10.67	0.34	0.054
N23	LP(1)	C11-C12	∂*	10.66	0.86	0.087
N23	LP(1)	C3-C4	∂*	10.36	0.86	0.085
C76-C78	∂	C78-N82	∂*	9.11	1.64	0.11
C11-N23	π	C14-C16	π*	9.04	0.33	0.051
C74-H75	∂	S69-C72	∂*	8.62	0.72	0.07
C78-N82	∂	C76-C78	∂*	8.16	1.56	0.102
C78-N82	π*	C74-C76	π*	8.14	0.36	0.051
C67-S69	∂	C63-C65	π*	0.51	0.69	0.018

Table S16: Second order Perturbation theory analysis of Fock matrix in NBO **Q4D1**

**Donor(i)**	**Type**	**Acceptor(j)**	**Type**	**E(2)**	**E(J)E(i)[b](a.u)**	**F(I,j)[e](a.u)**
C1-C2	∂	C2-C3	∂^*	4.10	1.39	0.067
C1-C6	π	C2-C3	π^*	36.93	0.34	0.100
C1-H7	∂	C2-C3	∂^*	5.15	1.19	0.070
C2-C3	π	C1-C6	π^*	34.11	0.34	0.097
C3-C8	∂	C34-C35	π^*	0.68	0.82	0.023
C4-C5	π	C1-C6	π^*	38.32	0.34	0.103
C4-N23	∂	C11-C14	∂^*	3.92	1.40	0.067
C5-H9	∂	C3-C4	∂^*	5.04	1.17	0.069
C6-H10	∂	C1-C6	∂^*	0.58	1.20	0.024
C8-C12	π	C11-N23	π^*	35.50	0.36	0.103
C12-H13	∂	C3-C8	∂^*	5.32	1.18	0.071
C14-C16	∂	C19-N24	∂^*	6.15	1.27	0.079
C14-C16	π	C11-N23	∂^*	0.72	0.92	0.025
C14-C16	π	C19-C21	π^*	29.38	0.36	0.095
C15-C17	π	C14-C16	π^*	30.30	0.36	0.096
C19-C21	∂	C29-C30	∂^*	5.51	1.38	0.078
C25-C30	∂	C25-C26	∂^*	4.92	1.36	0.073
C25-C30	π	C28-C29	π^*	34.72	0.35	0.100
C26-C27	∂	N24-C25	∂^*	6.35	1.29	0.081
C28-C29	π	C26-C27	π^*	29.98	0.36	0.094
C28-C29	π	C105-S107	∂^*	1.54	0.56	0.028
C34-C35	∂	C8-C12	π^*	1.07	0.84	0.028
C34-C35	∂	C37-N53	∂^*	6.04	1.27	0.078
C34-C36	∂	C8-C12	π^*	1.10	0.83	0.029
C36-C39	π	C34-C35	π^*	30.97	0.36	0.097
C37-C41	∂	C43-C45	π^*	5.41	1.37	0.077
C37-C41	π	C36-C39	π^*	31.65	0.36	0.097
C105-C106	∂	C28-C105	∂^*	5.09	1.35	0.074
C105-C106	π	C27-C28	∂^*	0.76	0.94	0.025
C105-C106	π	C28-C29	∂^*	0.63	0.95	0.023
C112-C113	π	C115-C117	π^*	30.86	0.37	0.098
C119-H120	∂	C121-C126	∂^*	7.41	1.16	0.083
C121-C126	∂	C126-N127	∂^*	8.89	1.76	0.112
C126-N127	π	C119-C121	∂^*	2.92	1.06	0.050
C126-N127	π	C121-C122	∂^*	3.45	0.89	0.050
N24	LP(1)	C19-C21	π^*	46.40	0.36	0.118
N24	LP(1)	C25-C30	π^*	49.92	0.35	0.122
N53	LP(1)	C37-C41	π^*	48.19	0.36	0.120
N53	LP(1)	C43-C44	π^*	47.31	0.36	0.119
O124	LP(2)	C122-O123	π^*	54.63	0.44	0.142

Table S17: Second order Perturbation theory analysis of Fock matrix in **Q4D2**

Donor(i)	Type	Acceptor(j)	Type	E(2)	E(J)E(i)[b](a.u)	F(I,j)[e](a.u)
C1-C2	∂	C2-C3	∂^*	4.10	1.39	0.067
C1-C2	π	C5-C6	π^*	25.12	0.39	0.089
C1-H7	∂	C2-C3	∂^*	5.15	1.19	0.070
C2-C3	∂	C3-C8	∂^*	4.94	1.35	0.073
C2-H54	∂	C3-C4	∂^*	4.96	1.17	0.068
C3-C4	π	C8-C12	π^*	25.51	0.36	0.091
C5-C6	π	C3-C4	π^*	24.80	0.36	0.089
C5-H9	∂	C3-C4	∂^*	5.04	1.17	0.069
C8-C12	∂	C34-C35	π^*	1.02	0.85	0.029
C8-C12	π	C11-N23	π^*	35.53	0.36	0.103
C8-C12	π	C34-C35	∂^*	1.71	0.95	0.038
C8-C12	π	C34-C36	∂^*	2.25	0.93	0.044
C11-N23	π	C3-C4	π^*	28.00	0.42	0.102
C14-C16	π	C11-N23	∂^*	0.72	0.92	0.025
C14-C16	π	C19-C21	π^*	29.39	0.36	0.095
C15-C17	π	C14-C16	π^*	30.29	0.36	0.096
C15-H18	∂	C14-C16	∂^*	5.14	1.20	0.070
C16-C19	∂	C14-C16	∂^*	4.72	1.41	0.073
C17-C21	∂	C15-C17	∂^*	3.78	1.42	0.066
C17-C21	∂	C21-C30	∂^*	5.92	1.35	0.080
C19-N24	∂	C16-C19	∂^*	2.67	1.49	0.056
C25-C30	π	C28-C29	π^*	34.93	0.35	0.100
C26-C27	∂	N24-C25	∂^*	6.34	1.29	0.081
C29-C30	∂	C21-C30	∂^*	6.00	1.36	0.081
C34-C35	∂	C8-C12	π^*	1.07	0.84	0.028
C34-C35	∂	C37-N53	∂^*	6.04	1.27	0.078
C34-C36	∂	C8-C12	π^*	1.10	0.83	0.029
C36-C39	π	C34-C35	π^*	30.97	0.36	0.097
C105-C106	π	C28-C29	∂^*	0.61	0.95	0.023
C115-C117	π	C119-C121	∂^*	36.66	0.37	0.104
C119-C121	∂	C122-N123	π^*	1.39	0.97	0.033
C121-C122	∂	C122-N123	∂^*	8.81	1.76	0.111
C121-C122	∂	C124-N125	π^*	2.69	0.97	0.046
C121-C124	∂	C122-N123	π^*	2.95	0.95	0.048
C121-C124	∂	C124-N125	∂^*	8.83	1.75	0.112
N24	LP(1)	C19-C21	π^*	46.31	0.36	0.118
N24	LP(1)	C25-C30	π^*	50.15	0.35	0.122
N53	LP(1)	C37-C41	π^*	48.20	0.36	0.120
N53	LP(1)	C43-C44	π^*	47.30	0.36	0.119
S114	LP(2)	C112-C113	π^*	35.48	0.34	0.098

Table S18: Second order Perturbation theory analysis of Fock matrix in **Q5D1**

**Donor (i)**	**Type**	**Acceptor (j)**	**Type**	**E (2)[a] [kJ/mol]**	**E(j)-E(i)[b] [a.u.]**	**F(i,j)c [a.u.]**
C_1-C_6	π	C_2-C_3	π^*	36.91	0.34	0.100
C_1-C_6	π	C_2-C_5	π^*	33.60	0.34	0.095
C_1-H_7	σ	C_2-C_3	σ^*	5.14	1.19	0.070
C_2-C_3	π	C_1-C_6	π^*	34.11	0.34	0.097
C_2-C_3	π	C_4-C_5	π^*	29.63	0.34	0.090
C_2-C_3	π	C_8-C_{12}	π	24.81	0.36	0.089
C_4-C_5	π	C_1-C_6	π^*	38.31	0.34	0.103
C_4-C_5	π	C_2-C_3	π^*	28.37	0.33	0.087
C_4-C_5	π	C_{11}-N_{23}	π^*	23.33	0.33	0.082
C_8-C_{12}	π	C_2-C_3	π^*	21.33	0.37	0.083
C_8-C_{12}	π	C_{11}-N_{23}	π^*	35.50	0.36	0.103
C_8-C_{12}	π	C_{34}-C_{35}	π^*	5.95	0.38	0.043
C_{11}- N_{23}	π	C_4-C_5	π^*	27.19	0.41	0.101
C_{11}- N_{23}	π	C_8-C_{12}	π^*	14.30	0.44	0.071
C_{11}- N_{23}	π	C_{14}-C_{16}	π^*	9.17	0.43	0.058
C_{12}- H_{13}	σ	C_3-C_8	σ^*	5.32	1.18	0.071
C_{14}-C_{16}	σ	C_{19}-N_{24}	σ^*	6.16	1.27	0.079
C_{25}-C_{30}	π	C_{28}-C_{29}	π^*	43.77	0.35	0.100
C_{26}-C_{27}	σ	N_{24}-C_{25}	σ^*	6.35	1.29	0.081
C_{36}-C_{39}	π	C_{34}-C_{35}	π^*	31.00	0.36	0.097
C_{37}-C_{41}	π	C_{36}-C_{39}	π^*	31.64	0.36	0.097
C_{43}-C_{44}	π	C_{45}-C_{47}	π^*	31.51	0.36	0.097
C_{14}-C_{16}	π	C_{15}-C_{17}	π^*	345.17	0.01	0.095
C_{19}-C_{21}	π	C_{15}-C_{17}	π^*	216.13	0.02	0.095
C_{25}-C_{30}	π	C_{26}-C_{27}	π^*	362.70	0.01	0.095
C_{25}-C_{30}	π	C_{28}-C_{29}	π^*	497.79	0.01	0.097
C_{37}-C_{41}	π	C_{36}-C_{39}	π^*	321.77	0.01	0.096
C_{43}-C_{44}	π	C_{45}-C_{47}	π	304.59	0.01	0.095
C_{124}.C_{125}	π	C_{120}-C_{122}	π^*	239.00	0.01	0.077
C_{45}-C_{47}	σ	C_{46}-C_{49}	σ^*	31.37	0.36	0.097
N_{23}	LP(1)	C_3-C_4	σ^*	11.32	099	0.095
N_{23}	LP(1)	C_{11}-C_{12}	σ^*	11.42	0.99	0.096
N_{24}	LP(1)	C_{19}-C_{21}	π^*	46.39	0.36	0.118
N_{24}	LP(1)	C_{25}-C_{30}	π^*	46.95	0.35	0.122
N_{24}	LP(1)	C_{58}-C_{79}	σ^*	6.88	0.74	0.070
N_{53}	LP(1)	C_{37}-C_{41}	π^*	48.20	0.36	0.120
N_{53}	LP(1)	C_{43}-C_{44}	π^*	47.36	0.36	0.119
N_{53}	LP(1)	C_{55}-C_{61}	σ^*	7.01	0.74	0.071
S_{119}	LP(2)	C_{117}-C_{118}	π^*	32.27	0.35	0.095
S_{119}	LP(2)	C_{120}-C_{122}	π^*	29.83	0.35	0.091
S_{126}	LP(2)	C_{124}-C_{125}	π^*	34.99	0.34	0.097
S_{126}	LP(2)	C_{127}-C_{129}	π^*	28.57	0.34	0.088
N_{135}	LP(1)	C_{132}-C_{134}	σ^*	11.63	1.15	0.103
O_{137}	LP(2)	C_{132}-C_{136}	σ^*	21.69	0.81	0.120
O_{137}	LP(2)	C_{136}-O_{138}	σ^*	36.89	0.73	0.148
O_{138}	LP(1)	C_{136}-O_{137}	σ^*	6.38	1.36	0.083

O_{138}	LP(2)	C_{136}-O_{137}	π^*	54.43	0.44	0.142

a)ED/e is the electron density of donor and acceptor of NBO orbitals.
b)E(2) means energy of hyperconjugative interaction (stabilization energy).
c)E(j)_E(i) is the energy difference between donor and acceptor I and j NBO orbitals.
d)F(i,j) is the Fock matrix element between I and j NBO orbitals.

Table S19: Wave length, excitation energy and oscillator strength of investigated compound **Q3.**

**NO**	**DFT λ (*nm*)**	**E(cm-1)**	***f***	**MO contributions**
1	320.4636796	31204.7843	1.2752	H-2→LUMO (68%)
2	319.4810168	31300.76428	0.2672	H-2→LUMO (10%), HOMO→LUMO (55%), HOMO→L+1 (20%)
3	305.8543874	32695.29689	0.1201	H-1→LUMO (36%), H-1→L+1 (24%), H-1→L+2 (29%)
4	295.7356002	33813.98788	1.0752	H-4→LUMO (12%), H-3→LUMO (54%), H-3→L+1 (11%)
5	282.8751837	35351.28062	0.1974	H-5→LUMO (20%), H-2→L+1 (45%)
6	271.9726962	36768.39675	0.0383	H-8→LUMO (81%)

MO=molecular orbital, H=HOMO, L=LUMO, *f*= oscillator strength

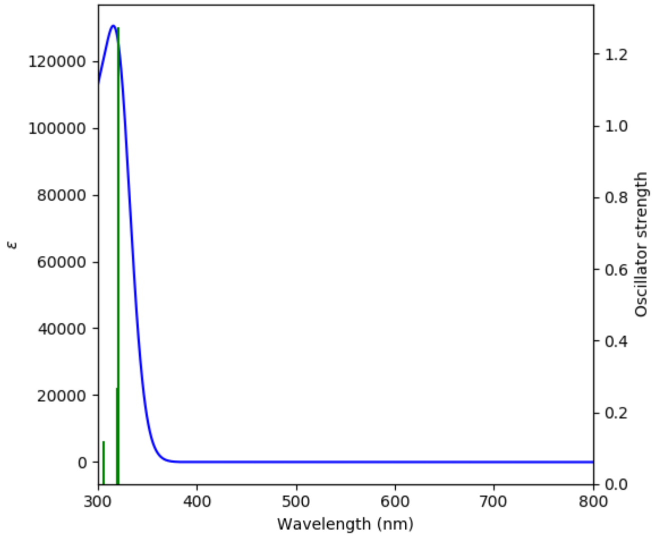

Figure S1: Graph of investigated compound **Q3**.

Table S20: Wave length, excitation energy and oscillator strength of investigated compound **Q4**

NO	DFT λ (*nm*)	E(cm-1)	*f*	MO contributions
1	315.68	31677.4	1.381	H-2→LUMO (76%)
2	289.066	34594.164	0.669	H-4→L (14%), H-3→L (54%), H-3→L+1 (9%), H-3→L+2 (5%), H-2→L (3%)
3	278.570	35897.565	0.197	H-5→L (19%), H-2→L+1 (41%), H-5→L+2 (2%), H-4→L (5%), H-4→L+3 (6%), H-3→L+1 (2%), H-2→L+2 (7%)
4	314.550	31791.368	0.056	H→L (59%), H→L+1 (25%), H-2→L+5 (3%), H→L+3 (5%)
5	302.066	33105.255	0.048	H-1→L (34%), H-1→L+1 (21%), H-1→L+2 (31%) H-3→L+4 (4%), H-2→L (3%)
6	268.594	37230.809	0.023	H-8→L (76%),H-8→L+2 (3%), H-6→L (4%), H-3→L+2 (2%)

MO=molecular orbital, H=HOMO, L=LUMO, *f*= oscillator strength

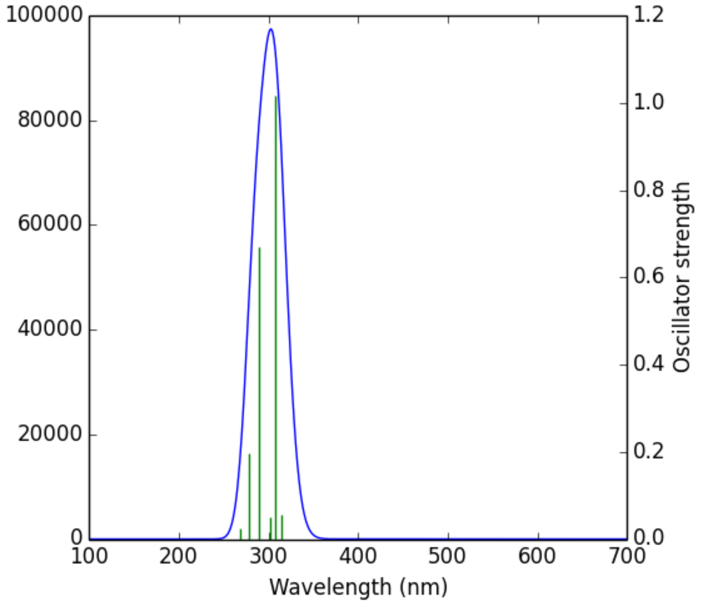

Figure S2: Graph of of investigated compound **Q4**

Table S21: Wave length, excitation energy and oscillator strength of investigated compound **Q5.**

**NO**	**DFT λ (*nm*)**	**E (cm-1)**	***f***	**MO contributions**
1	316.8520	31560.474	1.364	H-2→LUMO (75%)
2	288.306	34685.306	0.6462	H-4→L (16%), H-3→L (52%), H-3→L+1 (9%), H-3→L+2 (5%), H-2→L (2%)
3	279.021	35839.493	0.2133	H-5→L (19%), H-2→L+1 (42%), H-5→L+2 (2%), H-4→L (5%), H-4→L+3 (6%), H-3→L+1 (3%), H-2→L+2 (6%)
4	315.262	31719.585	0.0569	H→L (60%), H→L+1 (24%),H-2→L+5 (3%), H→L+3 (5%)
5	301.457	33172.199	0.0513	H-1→L (33%), H-1→L+1 (21%), H-1→L+2 (33%),H-3→L+4 (4%)
6	269.020	37171.930	0.0251	H-8→L (76%). H-8→L+2 (3%), H-6→L (4%), H-3→L+2 (2%)

MO=molecular orbital, H=H, L=Lumo, *f*= oscillator strength

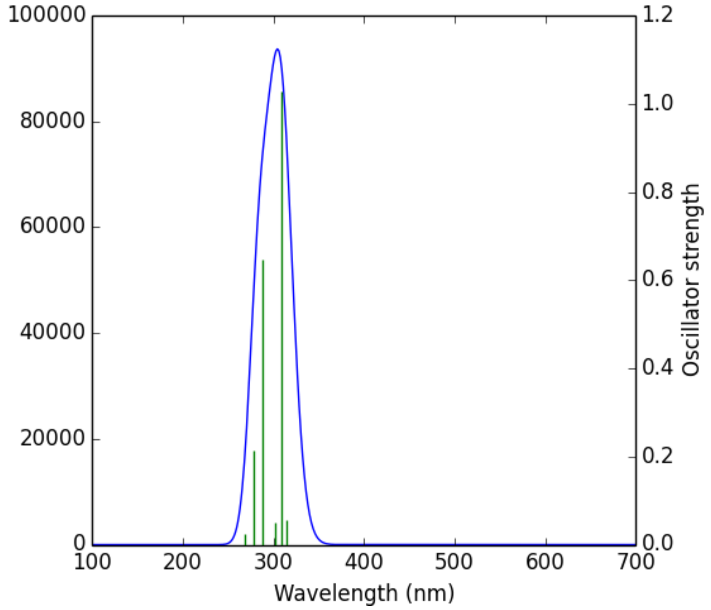

Figure S3: Graph of of investigated compound **Q5**.

Table S22: Wave length, excitation energy and oscillator strength of investigated compound **Q3D1**

NO	DFT λ (*nm*)	E(cm^{-1})	*f*	MO contributions
1	375.152	26655.817	0.925	H-4⟶L (33%), H-1⟶L (57%)
2	321.50	31103.965	1.5473	H-2→L+1 (77%) H-6→L+1 (4%), H-3→L
3	306.210	32657.388	0.006	H-4⟶L+1 (12%), H-1⟶L+1 (45%), H-1⟶L+2 (14%),H-4⟶L+2 (5%), H-2⟶L+7 (3%), H⟶L+1 (4%)
4	304.764	32812.247	0.048	H⟶L+1(33%),H⟶L+2(27%),H⟶L+4(24%)H-3⟶L+6 (4%), H-1⟶L+1 (4%)
5	298.887	33457.490	0.0006	H⟶L (90%) H-1⟶L (6%)
6	296.365	33742.204	0.015	H-4⟶L(45%),H-1⟶L(19%),H-1⟶L+3(16%)H-4⟶L+3 (2%), H⟶L (7%)

MO=molecular orbital, H=HOMO, L=LUMO, ***f*****=** oscillator strength

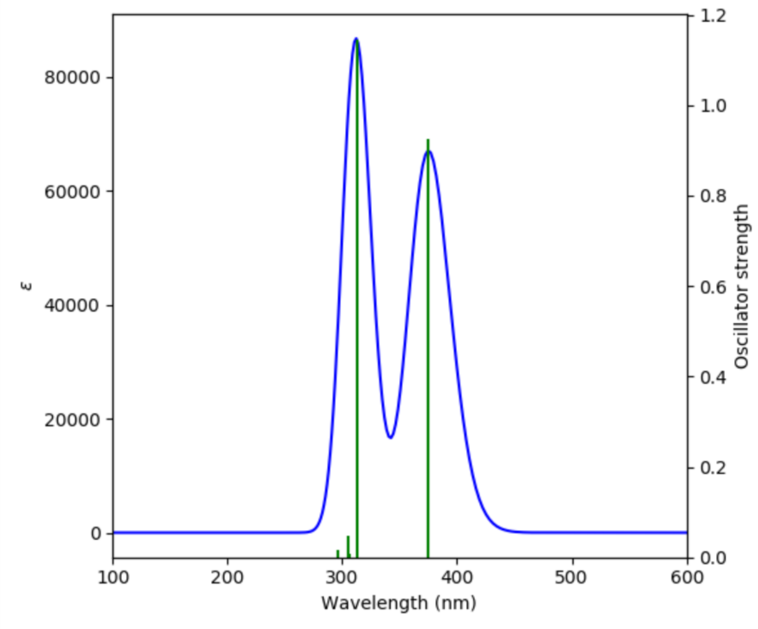

Figure S4: Graph of investigated compound **Q3D1**

Table S23: Wave length, excitation energy and oscillator strength of investigated compound **Q3D2**

NO	DFT λ (*nm*)	E(cm^{-1})	*f*	MO contributions
1	315.41	31701.62183	1.2703	H-3⟶L+1 (26%), H-2⟶L+1 (52%)
2	334.036	29936.881	0.001	H⟶L (98%)
3	317.225	31523.373	0.073	H-4⟶L(60%),H-1⟶L(17%),H-1⟶L+3(10%)H-9⟶L(3%)
4	307.944	32473.495	0.695	H-3⟶L+1(18%), H-2⟶L+1 (24%), H⟶L+1 (17%)H-6⟶L+1 (3%), H-2⟶L (7%), H⟶L+2 (8%), H⟶L+4 (9%)
5	307.356	32535.599	0.197	H-2⟶L+1 (18%), H⟶L+1 (26%), H⟶L+2 (15%), H⟶L+4 (14%) H-3⟶L+1 (8%), H-2⟶L (6%)
6	304.159	32877.578	0.035	H-1⟶L+1 (50%), H-1⟶L+2 (18%) H-4⟶L+1 (9%), H-4⟶L+2 (4%), H-2⟶L+1 (3%), H-1⟶L+5 (2%)

MO=molecular orbital, H=H, L=L, *f*= oscillator strength

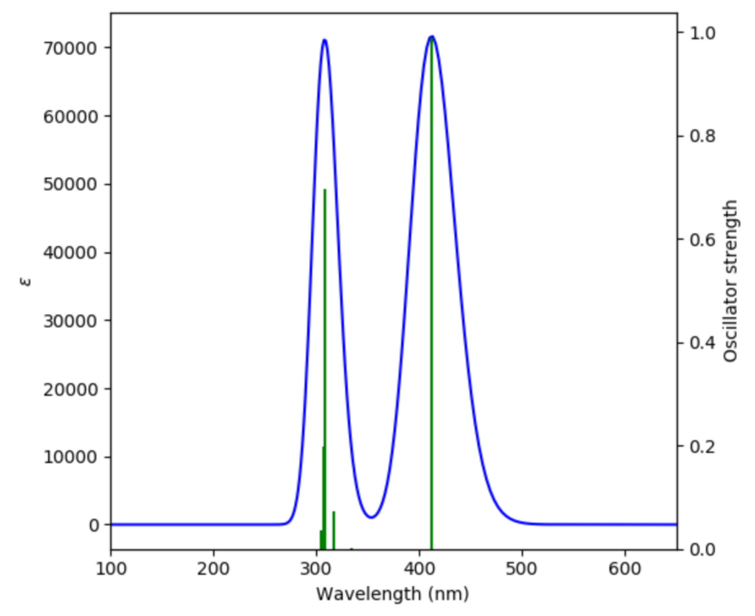

Figure S5: Graph of investigated compound **Q3D2**

Table S25: Wave length, excitation energy and oscillator strength of investigated compound **Q3D3**

NO	DFT *λ* (*nm*)	E(cm^{-1})	*f*	MO contributions
1	642.571	15562.468	0.0001	H⟶L (100%)
2	559.142	17884.538	0.002	H-1⟶L (99%)
3	524.645	19060.494	0.193	H-2⟶L (95%) H-3⟶L (3%)
4	515.012	19416.991	0.003	H-3⟶L (96%) H-2⟶L (3%)
5	416.319	24019.998	0.003	H-4⟶L (99%)
6	418.66	23885.30	1.206	H-4⟶LUMO (33%), H-1⟶LUMO (61%)

MO=molecular orbital, H=HOMO, L=LUMO, ***f***= oscillator strength

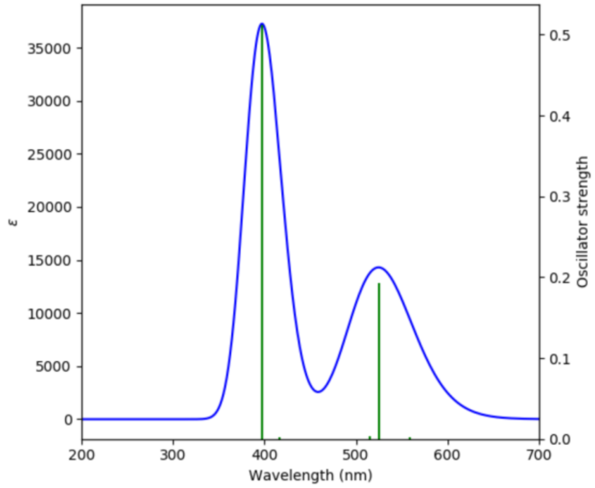

Figure S6: Graph of investigated compound **Q3D3**

Table S26: Wave length, excitation energy and oscillator strength of investigated compound **Q4D1**

NO	DFT *λ* (*nm*)	E(cm^{-1})	*f*	MO contributions
1	482.89	20708.28	1.8357	H-4⟶LUMO (17%), HOMO⟶LUMO (76%)
2	309.517	32308.373	0.852	H-3→L+1 (12%), H-2→L+1 (62%),H-7→L+1 (2%), H-3→L+3 (3%), H-2→L+3 (3%)
3	295.797	33806.962	0.821	H-5→L+1 (11%), H-3→L+1 (47%), H-2→L+1 (13%), H-3→L+3 (6%), H-3→L+4 (3%), H-2→L+3 (5%)
4	329.042	30391.180	0.111	H-4→L+1 (11%), H→L+1 (43%), H→L+3 (17%), H-4→L (5%), H-4→L+3 (3%), H→L+2 (4%), H→L+5 (4%)
5	306.252	32652.775	0.037	H-1→L+1 (40%), H-1→L+3 (21%), H-1→L+4 (26%), H-3→L+7 (4%)
6	318.690	31378.410	0.034	H-4→L (40%), H→L+2 (20%), H-7→L (5%), H-6→L (7%), H-3→L (2%), H-2→L (4%), H→L (6%), H→L+3 (4%)

MO=molecular orbital, H=HOMO, L=LUMO, *f*= oscillator strength

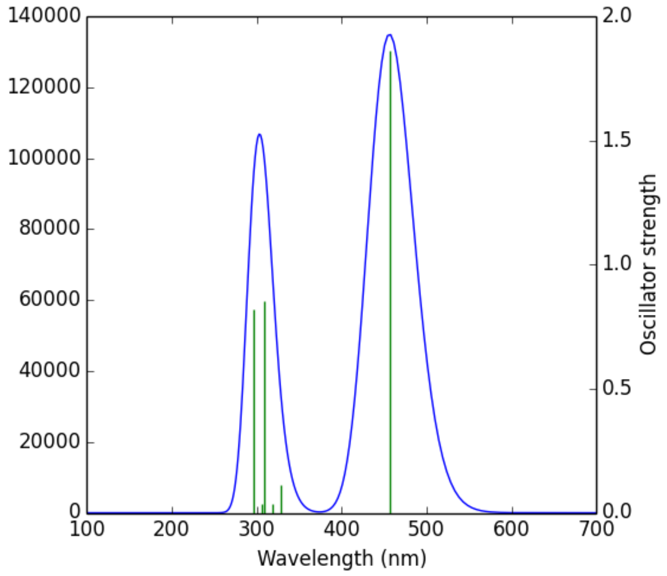

Figure S7: Graph of investigated compound **Q4D1**

Table S27: Wave length, excitation energy and oscillator strength of investigated compound **Q4D2**

NO	DFT ***λ (nm)***	E(cm-1)	*f*	MO contributions
1	497.109	20116.27	1.8506	H-4⟶LUMO (17%), HOMO⟶LUMO (76%)
2	309.548	32305.147	0.805	H-3→L+1 (13%), H-2→L+1 (59%), H-7→L+1 (2%), H-3->L+3 (3%), H-2→L (2%), H-2→L+3 (3%)
3	297.907	33567.414	0.134	H-3→L (14%), H-2→L (50%) H-6→L (3%), H-4→L+1 (3%), H-2→L+1 (4%), H-2→L+2 (4%), H→L+1 (4%), H→L+2 (4%)
4	329.523	30346.82	0.093	H-4→L (12%), H→L+1 (37%), H→L+3 (14%), H-6→L (2%), H-4→L+1 (9%), H-4→L+3 (3%), H→L+2 (6%), H→L+5 (3%)
5	323.572	30904.959	0.046	H-4→L (38%), H→L+2 (12%), H-7→L (5%), H-6→L (6%), H-3→L (3%), H-2→L (6%), H→L (7%), H→L+1 (7%), H→L+3 (6%)
6	306.290	32648.742	0.038	H-1→L+1 (40%), H-1→L+3 (22%), H-1→L+4 (26%), H-3→L+8 (4%)

MO=molecular orbital, H=HOMO, L=LUMO, *f*= oscillator strength

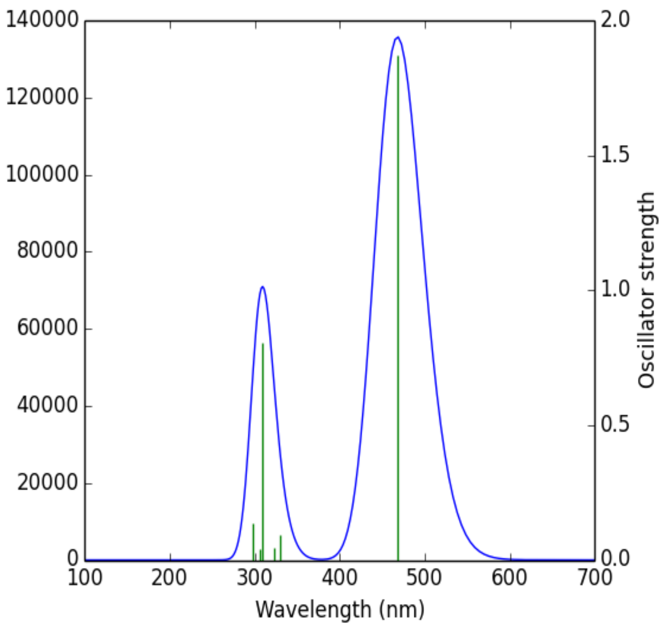

Figure S8: Graph of investigated compound **Q4D2**

Table S28: Wave length, excitation energy and oscillator strength of investigated compound **Q5D1**

**NO**	**DFT λ (*nm*)**	**E(cm-1)**	***f***	**MO contributions**
1	484.351	20646.18	1.713	H-4⟶LUMO (16%), HOMO⟶LUMO (77%)
2	309.308	32330.151	0.899	H-3→L+1 (12%), H-2→L+1 (61%), H-7→L+1 (2%), H-3→L+3 (2%), H-2→L+3 (3%)
3	295.522	33838.418	0.783	H-5→L+1 (11%), H-3→L+1 (48%), H-2→L+1 (12%), H-3->L+3 (5%), H-3→L+4 (4%), H-2→L+3 (4%)
4	328.763	30416.990	0.150	H-4→L+1 (11%), H→L+1 (43%), H→L+3 (12%), H-4→L (4%), H-4→L+3 (3%), H-2→L+3 (2%), H→L+2 (8%), H→L+5 (4%)
5	306.146	32664.066	0.038	H-1→L+1 (40%), H-1→L+3 (17%), H-1→L+4 (26%), H-3→L+7 (4%), H-2→L+1 (2%), H-1→L+2 (5%)
6	318.575	31389.702	0.008	H-4→L (45%), H→L+2 (10%), H-7→L (5%), H-6→L (7%), H-3→L (3%), H-2→L (5%), H→L (7%), H→L+3 (7%)

MO=molecular orbital, H=HOMO, L=LUMO, *f*= oscillator strength

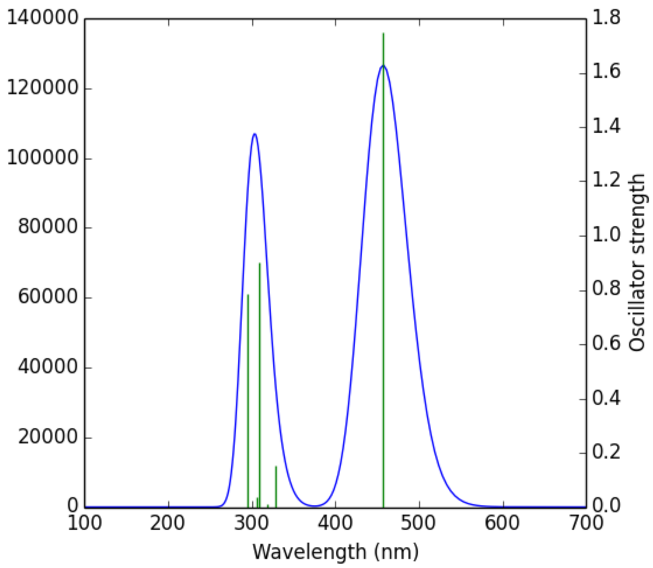

Figure S9: Graph of investigated compound **Q5D1**
